# Impact of Long COVID on productivity and informal caregiving

**DOI:** 10.1007/s10198-023-01653-z

**Published:** 2023-12-26

**Authors:** Joseph Kwon, Ruairidh Milne, Clare Rayner, Román Rocha Lawrence, Jordan Mullard, Ghazala Mir, Brendan Delaney, Manoj Sivan, Stavros Petrou

**Affiliations:** 1 Nuffield Department of Primary Care Health Sciences, University of Oxford, Oxford, England; 2 Public Health, Wessex Institute, University of Southampton, Southampton, England; 3 Locomotion Patient Advisory Group (Co-Lead), University of Leeds, Leeds, England; 4 ELAROS 24/7 Ltd, Sheffield, England; 5 School of Medicine, University of Leeds, Leeds, England; 6 Department of Sociology, University of Durham, Durham, England; 7 Department of Surgery and Cancer, Imperial College London, London, England; 8 Academic Department of Rehabilitation Medicine, University of Leeds, Leeds, England

**Keywords:** Long COVID, Productivity, Informal caregiver burden, Health economic evaluation, I10

## Abstract

**Background:**

Around 2 million people in the UK suffer from Long COVID (LC). Of concern is the disease impact on productivity and informal care burden. This study aimed to quantify and value productivity losses and informal care receipt in a sample of LC patients in the UK.

**Methods:**

The target population comprised LC patients referred to LC specialist clinics. The questionnaires included a health economics questionnaire (HEQ) measuring productivity impacts, informal care receipt and service utilisation, EQ-5D-5L, C19-YRS LC condition-specific measure, and sociodemographic and COVID-19 history variables. Outcomes were changes from the incident infection resulting in LC to the month preceding the survey in paid work status/h, work income, work performance and informal care receipt. The human capital approach valued productivity losses; the proxy goods method valued caregiving hours. The values were extrapolated nationally using published prevalence data. Multilevel regressions, nested by region, estimated associations between the outcomes and patient characteristics.

**Results:**

366 patients responded to HEQ (mean LC duration 449.9 days). 51.7% reduced paid work hours relative to the pre-infection period. Mean monthly work income declined by 24.5%. The average aggregate value of productivity loss since incident infection was £10,929 (95% bootstrap confidence interval £8,844-£13,014) and £5.7 billion (£3.8-£7.6 billion) extrapolated nationally. The corresponding values for informal caregiving were £8,726 (£6,247-£11,204) and £4.8 billion (£2.6-£7.0 billion). Multivariate analyses found significant associations between each outcome and health utility and C19-YRS subscale scores.

**Conclusion:**

LC significantly impacts productivity losses and provision of informal care, exacerbated by high national prevalence of LC.

**Supplementary Information:**

The online version contains supplementary material available at 10.1007/s10198-023-01653-z.

## Background

The term ‘Long COVID’ (LC) covers two conditions: (i) ‘ongoing symptomatic COVID-19’ for signs and symptoms of acute COVID-19 between 4 and 12 weeks after the incident COVID-19 infection; and (ii) ‘post-COVID-19 syndrome’ for signs and symptoms beyond 12 weeks not explained by an alternative diagnosis [1]. As of 5th March 2023, around 1.9 million people in the UK had self-reported LC, 41% of whom self-reported the condition 2 years or more after the incident COVID-19 infection [2]. Symptoms of LC include fatigue, breathlessness, pain, neurocognitive dysfunction, exercise intolerance, and functional disability [3–6]. The condition is characterised by clusters of symptoms that change over time, including fluctuations in severity and sudden relapses [1, 7].

Of particular concern is the impact of LC on work status and productivity of patients and, in turn, on the national workforce. The 2022 All-Party Parliamentary Group report on Long COVID, for example, estimated that the 219 NHS trusts in England lost around 1.8 million working days to LC absences between March 2020 and September 2021 [8]. The report goes on to classify LC as an occupational disease and calls for the establishment of a compensation scheme for key frontline workers (e.g. healthcare workers, teachers) who contract the disease [8]. The substantial impact of LC on productivity has likewise been confirmed by numerous UK and non-UK studies of general or general working populations [9–28] and specific professions including healthcare workers, teachers, and athletes [29–33]. Productivity in employment not only increases national income, but is also a determinant of health and well-being [34, 35]. Productivity loss due to sickness begets further ill health via mediators including financial instability [36], lack of cognitive stimulation [37], and loss of social identity [38]. Voluntary activities, particularly by retired older persons, likewise contributes to local and wider communities [39]. It is therefore of national and individual interest that LC care strategies place adequate emphasis on vocational rehabilitation to help LC patients return to work or usual activities [40].

Of equal concern is the informal care burden on family members and other caregivers imposed by LC, which has not been a subject of recent research, even as the impact of the COVID-19 pandemic on informal caregivers in general becomes better known [41]. It is important to value the ‘invisible’ contributions of informal caregivers in providing services, which would otherwise result in unmet care needs, incur substantial private care expenditures, and/or place additional strains on the health and social care sectors [42, 43]. A recent study set in Italy, for example, has shown that increased availability of female informal caregivers in a region is negatively associated with the level of public expenditure on long-term care [44]. Productivity losses are incurred when caregivers reduce or give up their employment [42]. Moreover, informal caregivers are often ill-equipped to handle the care burden associated with complex multimorbidity and/or they themselves may be frail and at risk of emotional and physical burnout from caregiving [45, 46]. Overall, there is a strong rationale for the inclusion of productivity and informal caregiver impacts within cost-of-illness studies and economic evaluations to account for the full range of costs incurred by the disease and alleviated by prevention and/or care strategies [42, 47–49].

LOng COvid Multidisciplinary consortium Optimising Treatments and servIces acrOss the NHS (LOCOMOTION) is a mixed-methods study that aims to optimise all aspects of LC care in the UK [50]. A core objective of LOCOMOTION is to promote the vocational rehabilitation of LC patients. Accordingly, the economic evaluation planned for the study will adopt a societal perspective to consider how specialist LC clinics impact productivity losses and informal caregiver burden. Such evaluation requires reliable data on the productivity and caregiving impacts of the condition. So would any further evaluations outside of LOCOMOTION that develop decision-analytic models to examine the cost-effectiveness of prevention and care strategies targeting acute and/or Long COVID [51–53]. Monetary values concerning the productivity and caregiving impacts, stratified by factors such as age group, sex, and duration of LC, would help parameterise such models [54–56]. They also offer a resource for extrapolating from the study sample to the wider LC population. None of the previous studies of LC productivity loss [9–33], however, have estimated its monetary value, presenting a gap in the literature. Likewise, none has estimated the economic value of informal caregiving attributable to LC.

Moreover, associations between the type and magnitude of productivity loss and caregiving impact and sociodemographic (e.g. socioeconomic deprivation, ethnicity) and health-related (e.g. duration of LC since the incident infection) factors should clarify where adverse impacts are concentrated and focus policy and care attention accordingly. Likewise, associations between productivity and caregiving impacts and validated patient-reported outcome measures (PROMs) such as the COVID-19 Yorkshire Rehabilitation Scale (C19-YRS), the first validated PROM for LC [3, 57, 58], should facilitate rehabilitation planning for LC patients, clinicians, and employers.

Therefore, this study aims to describe, measure, and value productivity losses and informal care receipt among LC patients referred to specialist LC care clinics as part of LOCOMOTION. The objectives are to: present the productivity and caregiving impacts for a cross-sectional sample of patients and across its subgroups; estimate the monetary value of those impacts; and identify patient characteristics that are associated with those impacts.

## Methods

Ethics approval for the LOCOMOTION study was obtained from the Bradford and Leeds Research Ethics Committee on behalf of Health Research Authority and Health and Care Research Wales on 6th January 2022 (reference: 21/YH/0276) [50]. Patients provided informed consent for research participation, patient-reported data collection, data analysis, and research publication through the ELAROS digital system (https://www.elaros.com/) when they enrolled into the study and registered on the system.

### Target population and data collection

The target population comprised LC patients newly referred to specialist LC clinics participating in the LOCOMOTION study and subsequently registered on the ELAROS system. The cross-sectional sample drawn for this particular study comprised newly referred patients recruited between 8th August 2022 (when the health economic questionnaire went live on the ELAROS system) and 13th February 2023, a recruitment period of six months. The ELAROS system includes several digital patient-reported outcome measures (DPROMs), administered using the app- and web-based interfaces within ELAROS.

### Health economics questionnaire (baseline version)

The health economics questionnaire (HEQ) contained questions on: (i) service(s) received at initial contact with the specialist LC clinic (phone/online consultation, face-to-face doctor consultation, physiotherapy, occupational therapy, speech and language therapy, fatigue management, counselling, peer support network, dietitian, welfare advice, multidisciplinary session, other) and private expenses; (ii) secondary, community health and social service utilisation, medications prescribed, and private care expenditures for LC symptoms in the month preceding the survey (henceforth, ‘previous month’); (iii) productivity in the period prior to the incident COVID-19 infection that resulted in LC (henceforth, ‘pre-infection period’) and in the previous month (further described below); and (iv) informal care receipt in the previous month (further described below). The HEQ was co-developed with lead patient advisory group members (RM, CR, Ms Karen Cook) and piloted on further patient advisory group members (*N *= 8). The time to completion ranged between 15 and 25 min. The full HEQ is available in the Supplementary Material.

### Outcomes related to productivity and informal care receipt

Table 1 lists the outcomes related to productivity and informal care receipt analysed in this study, which were generated from the relevant questions included in the HEQ. The HEQ question D1 also provided free-text space for participants to describe any other ways in which LC symptoms had affected their work status. This generated qualitative data on productivity impacts and, where available, helped internally validate the productivity outcomes. Specifically, counterintuitive responses were identified (e.g. participant described having moved from full- to part-time work, but reported higher weekly work hours), which were subsequently counted as missing and dropped from analysis.

**Table 1:** Outcomes related to productivity and informal care receipt

Outcome	Variable type	Note
(A) Change in weekly paid work status/h	Categorical variable: (1) Reduced paid work hours in the previous month compared to the pre-infection period (2) No longer engaged in paid work (i.e. no paid work hours) and no work income over the previous month due to lost employment, early retirement, or other reason that was not stated. Where a respondent provided work hours for the pre-infection period but left the response for the previous month empty, it was assumed that no hours were worked in the previous month (3) No longer engaged in paid work but receiving work income over the previous month through long-term sick leave arrangement or for other reason that was not stated (4) No change in paid work hours in the previous month compared to the pre-infection period (5) Increased paid work hours in the previous month compared to the pre-infection period (6) Already retired or not engaged in paid work (e.g. studying, engaged in unpaid work only, unemployed) in the pre-infection period and the same status in the previous month (7) Not engaged in paid work due to pre-existing sickness or disability in the pre-infection period and the same status in the previous month (8) Unclear	See questions D0 and D1 in HEQ. Monthly work income reported by participants was used to distinguish between categories (2) and (3)
(B) Return to pre-infection paid work status/h	Binary variable: (1) Did not return to pre-infection paid work status/h – categories (1), (2), or (3) for outcome (A) above (2) Returned to pre-infection paid work status/h – categories (4) or (5) for outcome (A) above	Derived from (A). Categories (6)–(8) in (A) counted as missing
(C) Change in weekly paid work hours	Continuous variable: Change in weekly paid work hours among those who were in paid employment in the pre-infection period (i.e. categories (1)–(5) for outcome (A) above)	Derived from (A). Categories (6)–(8) in (A) counted as missing
(D) Change in weekly unpaid work status/h	Categorical variable: (1) Reduced unpaid work hours (2) Stopped unpaid work (3) No change in (non-zero) unpaid work hours from the pre-infection period (4) Increased unpaid work hours	See questions D0 and D1 in HEQ
(E) Change in work performance	Continuous variable (−100 to 100): How health problem affected work performance scored on a scale from 0 (very little) to 100 (profoundly) in the pre-infection period and in the previous month. Change in work performance on a scale from −100 (maximum improvement) to 100 (maximum decline)	See questions D0 and D1 in HEQ
(F) Change in monthly work income	Continuous variable: Monthly work income in the pre-infection period and in the previous month. Change in monthly work income derived by subtraction	See questions D0 and D1 in HEQ. Stated income above £10,000 was assumed to be annual and divided by 12
(G) Receipt of any informal care	Binary variable: Yes/No	See question C1 in HEQ
(H) Relation to caregiver(s)	Free-text response: Relationship with up to three caregivers	See question C1 in HEQ
(I) Informal care help type	Free-text response: Help type received from up to three caregivers	See question C1 in HEQ
(J) Weekly hours of informal care	Continuous variable (≥ 0): Informal care hours received from up to three caregivers	See question C1 in HEQ
(K) Whether caregiver reduced work hour	Binary variable: Yes/No	See question C2 in HEQ
(L) Work hours reduced by caregiver	Continuous variable (≥ 0): Paid and/or unpaid work hours reduced by up to three caregivers	See question C2 in HEQ

*HEQ* health economics questionnaire

### Covariates

As noted above, the HEQ contained variables other than the outcomes related to productivity and informal care receipt: e.g. secondary, community health, and social service utilisation. These served as covariates in the multivariate analyses described below. The following variables were likewise collected digitally in ELAROS (using separate questionnaires from the HEQ) and served as further covariates:

#### EQ-5D-5L

The EQ-5D-5L health-related quality of life measure covers five dimensions, namely mobility, self-care, usual activities, pain and discomfort, and anxiety and depression, each with five levels of severity [59]. These are used to measure the respondent’s health ‘today’, i.e. the day of survey response. No EQ-5D-5L preference-based value set is currently recommended in the UK to convert the EQ-5D-5L dimension responses into health utility scores. The dimension responses were therefore converted into EQ-5D-3L (an earlier version of the EQ-5D with three response levels for each of the five dimensions) utility scores using an established algorithm [60]. The utility scores are anchored on a scale where 0 = dead and 1 = full health or no problem on all dimensions; utility scores below 0 are available to describe health states deemed worse than death. The EQ-5D-5L health status descriptive system is supplemented by the EQ-VAS, which measures the respondent’s self-rated health on a vertical visual analogue scale (range 0–100) [59].

#### COVID-19 Yorkshire Rehabilitation Scale (C19-YRS/m)

The C19-YRS is the first validated PROM in the literature for LC symptom type and severity [57]. Its 22 items produce four subscale scores: (i) symptom severity (range 0–100, higher score more severe); (ii) functional disability (0–50, higher score more disabled); (iii) overall health (0–10, higher score better health); and (iv) other symptoms (0–60, higher score more severe). The modified version (C19-YRSm) [3] has 17 items and a 4-point (rather than 11-point) response category for the items related to symptom severity and functional disability. It has the same four subscales as the original version but score ranges of 0–30, 0–15, 0–10, and 0–25, respectively. For subscales (i)–(iii) in both versions, participants were asked to describe their state ‘now’ and their state in the pre-infection period by recall. This enabled the measurement of changes to these subscale scores from the pre-infection period to the survey date.

For multivariate analyses (see below), a binary variable was created for each subscale to indicate participants who were in the highest quartile for the score change (score level for (iv)). The variables thus indicated quartiles who experienced the highest increases in symptom severity, the highest increases in functional disability, the best improvements in overall health (or the least decline in it), and the highest levels of other symptoms, respectively.

#### Sociodemographic and COVID-19 history variables

Participants reported their sociodemographic characteristics and COVID-19 history, including: age (integer in years), sex (male, female), ethnicity (White, Asian, Black, mixed, or other), region (Birmingham, Cardiff, Hertfordshire, Leeds, Leicester, London, Newcastle, Oxford, or Salford), postcode from which area-based index of multiple deprivation (IMD) decile was derived [61], occupation (free-text response, subsequently categorised by the 2007 UK Standard Industry Classification (SIC) codes [62]), date(s) of COVID-19 infection(s), whether a given infection led to LC (i.e. the incident infection, from the date of which the duration of LC was derived), positive COVID-19 test history, history of hospitalisation(s) due to acute COVID-19, history of intensive care unit (ICU) admission(s) due to acute COVID-19, and COVID-19 vaccination receipt(s) and their date(s) and brand(s).

### Analytic methods

Descriptive statistics for each outcome were estimated. Monetary values of productivity losses and informal care receipt were then estimated. For productivity losses, the human capital approach was used for valuation wherein each paid work hour loss was valued at the hourly wage [47]. For participants that reported their monthly work income and weekly paid work hours in the pre-infection period, this information was used to estimate their pre-infection hourly wage, assuming constant weekly work hours and four working weeks over the income-earning month. For those not reporting their pre-infection monthly income, this was predicted using linear regression adjusted for age, sex, IMD quintile, industry code, and region, with coefficients bootstrapped over 1,000 replications. To estimate the aggregate loss over the whole duration of LC, it was assumed that the weekly loss remained constant from the date of incident infection to the survey date. The mean aggregate losses bootstrapped over 1,000 replications were then reported by subgroups of sex, age group, ethnicity, IMD quintile, duration of LC, and industry group – (i) health and education, (ii) financial, information and communication technology (ICT), and professional, and (iii) other – alongside their respective bootstrapped standard error (SE) and 95% confidence interval (CI). Separate mean values were calculated across the subset who had been engaged in paid work in the pre-infection period and across the whole sample. The bootstrapped mean monthly losses were likewise reported.

For informal care, each hour of informal caregiving was valued using the proxy goods method [42]: i.e. it was assumed that in the absence of informal care, individuals would purchase private care as a substitute at the average hourly cost of £20 [63]. Like productivity impacts, the aggregate value of informal care was estimated by assuming the weekly cost remained constant from the date of incident infection. For those who reported receiving any informal care but did not specify the hours of informal care received, the latter was predicted using linear regression adjusted for IMD quintile and EQ-5D-3L utility score (there was no significant association between care hours and other potential covariates such as age, sex, and C19-YRS(m) subscales), with bootstrapped coefficients. The bootstrapped mean aggregate and monthly values were reported by sex, age group, ethnicity, IMD quintile, and duration of LC. Separate mean values were calculated across the subset who received any informal care and across the whole sample.

Economic values of productivity losses and informal care receipt were extrapolated to the national level in the UK using the LC prevalence data provided by the Office for National Statistics (ONS) [2]. Specifically, as of 5th March 2023, 381,000 individuals in the UK self-reported having LC symptoms that impacted their daily activities ‘a lot’. It was assumed that the study sample of LC patients referred to LC specialist clinic is representative of this national sub-population with severe disability due to LC. Of this sub-population, 175,000 individuals reported contracting the incident COVID-19 infection at least 2 years previously, 90,000 between 1 and 2 years previously, 99,000 less than 1 year previously, and 17,000 of unknown LC duration [2]. The bootstrapped mean values (for the whole study sample) by LC duration were applied to these prevalence estimates to estimate the national level impacts; those with unknown LC duration were not included in the extrapolation.

Multilevel logistic regressions, with participants nested by region, were estimated to identify factors significantly associated with the binary outcomes (B) and (G) in Table 1, namely whether the participant returned to pre-infection paid work status/h and whether the participant received any informal care. Multilevel linear regressions were likewise estimated for continuous outcomes (C), (E), and (F) in Table 1, namely change in paid work hours, change in work performance, and change in monthly income, respectively.

All regressions adjusted for duration of LC. In addition, four covariate sets of explanatory variables were used for each regression:

1. Demographic variables – sex, age group, ethnicity (white vs. minority ethnic), and IMD quintile.
2. COVID-19-related variables – hospitalisation for acute infection, single- vs. double-vaccinated, secondary and primary/social care use for LC symptoms in the previous month, and number of services receiving at initial LC clinic contact – plus variables in covariate set (1).
3. PROMs – EQ-5D-3L utility score multiplied by 100 for greater granularity (the coefficient now represents the impact of a score change of magnitude 0.01) and the quartiles of C19-YRS(m) subscale scores defined above – plus variables in covariate set (2).
4. Variables in covariate set (3) that had *P* values less than 0.1 for their coefficients being significantly different from zero.

Where the EQ-5D-3L utility score was included as an explanatory variable in covariate set (4), additional regressions were estimated with individual EQ-5D-5L dimension responses as explanatory variables instead of the EQ-5D-3L utility score. It was hypothesised that there are statistically significant and positive associations between higher severity of LC as measured by the PROMs (e.g. lower EQ-5D-3L utility score) and magnitude of the disease impacts on productivity and informal caregiving. All statistical analyses were implemented in STATA version 17 [64].

## Results

There were 714 new registrations on the ELAROS system from 9th August 2022 to 14th February 2023. Of these, 366 (51.3%) completed the HEQ and constituted the study sample. There was no statistically significant difference between the respondents and non-respondents to the HEQ in terms of mean age, sex, ethnicity (white vs. minority ethnic), IMD quintile, and hospitalisation due to acute COVID-19: see Table A1 in the Supplementary Material. See also “Discussion” for issues around representativeness of the study sample to the general LC patient population (comprising LC patients who are yet to be diagnosed and/or referred to LC specialist clinics) in the UK in terms of its ethnic and socioeconomic mix.

### Sample characteristics

Table 2 describes the sample characteristics, delineated by duration of LC in terms of the number of years since the incident COVID-19 infection that resulted in LC. Table A2 in the Supplementary Material reports the EQ-5D-5L dimension responses by duration of LC. Only a minority of the sample (10.9%) reported having been hospitalised for acute COVID-19.

**Table 2:** Sample characteristics by duration of Long COVID

	LC duration < 1 year (*N* = 163)	LC duration 1–2 years (*N* = 101)	LC duration > 2 years (*N* = 61)	LC duration unknown (*N* = 41)	Total (*N* = 366)
Male *N* (%)	50 (30.7)	35 (34.7)	23 (37.7)	10 (24.4)	118 (32.2)
Mean age (SD)	48.0 (12.3)	48.0 (11.7)	50.9 (11.0)	46.6 (10.4)	48.3 (11.7)
Ethnicity *N* (%)					
White	138 (84.7)	78 (77.2)	52 (85.2)	20 (48.8)	288 (78.7)
Minority ethnic	14 (8.6)	13 (12.9)	4 (6.6)	8 (19.5)	39 (10.7)
Missing	11 (6.7)	10 (9.9)	5 (8.2)	13 (31.7)	39 (10.7)
Region *N* (%)					
Birmingham	12 (7.4)	9 (8.9)	2 (3.3)	4 (9.8)	27 (7.4)
Cardiff	16 (9.8)	8 (7.9)	13 (21.3)	6 (14.6)	43 (11.8)
Hertfordshire	12 (7.4)	10 (9.9)	4 (6.6)	0 (0.0)	26 (7.1)
Leeds	25 (15.3)	25 (24.8)	9 (14.8)	5 (12.2)	64 (17.5)
Leicester	33 (20.2)	16 (15.8)	8 (13.1)	9 (22.0)	66 (18.0)
London	5 (3.1)	3 (3.0)	5 (8.2)	1 (2.4)	14 (3.8)
Newcastle	4 (2.5)	13 (12.9)	9 (14.8)	2 (4.9)	28 (7.7)
Oxford	34 (20.9)	6 (5.9)	1 (1.6)	5 (12.2)	46 (12.6)
Salford	22 (13.5)	11 (10.9)	10 (16.4)	9 (22.0)	52 (14.2)
IMD quintile *N* (%)					
Most deprived	21 (12.9)	16 (15.8)	8 (13.1)	0 (0.0)	45 (12.3)
2nd	20 (12.3)	12 (11.9)	6 (9.8)	1 (2.4)	39 (10.7)
3rd	14 (8.6)	12 (11.9)	9 (14.8)	0 (0.0)	35 (9.6)
4th	28 (17.2)	12 (11.9)	8 (13.1)	2 (4.9)	50 (13.7)
Least deprived	31 (19.0)	15 (14.9)	4 (6.6)	1 (2.4)	51 (13.9)
Missing	49 (30.1)	34 (33.7)	26 (42.6)	37 (90.2)	146 (39.9)
Industry of occupation *N* (%)					
Health and social work	29 (17.8)	19 (18.8)	20 (32.8)	9 (22.0)	77 (21.0)
Education	19 (11.7)	12 (11.9)	5 (8.2)	3 (7.3)	39 (10.7)
Professional	19 (11.7)	13 (12.9)	6 (9.8)	2 (4.9)	40 (10.9)
Administrative	12 (7.4)	9 (8.9)	0 (0.0)	1 (2.4)	22 (6.0)
Financial and ICT^a^	4 (2.5)	5 (5.0)	2 (3.3)	2 (4.9)	13 (3.6)
Public administration and defence	6 (3.7)	2 (2.0)	1 (1.6)	2 (4.9)	11 (3.0)
Accommodation, transport and trade^b^	7 (4.3)	10 (9.9)	1 (1.6)	1 (2.4)	19 (5.2)
Arts, student, and other industries^c^	12 (7.4)	3 (3.0)	2 (3.3)	1 (2.4)	18 (4.9)
Missing	55 (33.7)	28 (27.7)	24 (39.3)	20 (48.8)	127 (34.7)
Hospitalised for COVID-19 *N* (%)	10 (6.1)	23 (22.8)	6 (9.8)	1 (2.4)	40 (10.9)
Mean hospitalisation length in days (SD)	14.9 (27.9)	8.8 (9.8)	3.5 (5.2)	20.0 (N/A)	9.8 (15.9)
ICU admission for COVID-19 *N* (%)	3 (1.8)	6 (5.9)	0 (0.0)	1 (2.4)	10 (2.7)
Mean ICU admission length in days (SD)	11 (11.1)	2 (1.5)	N/A	1 (N/A)	4.6 (7.0)
COVID-19 vaccination *N* (%)					
Double vaccinated	89 (54.6)	51 (50.5)	30 (49.2)	0 (0.0)	170 (46.4)
Single vaccinated	28 (17.2)	23 (22.8)	13 (21.3)	3 (7.3)	67 (18.3)
Missing	46 (28.2)	27 (26.7)	18 (29.5)	38 (92.7)	129 (35.2)
Mean EQ-5D-3L utility (SD) [N]	0.515 (0.265) [*N* = 147]	0.508 (0.299) [*N* = 87]	0.398 (0.315) [*N* = 55]	0.578 (0.274) [*N* = 36]	0.501 (0.287) [*N* = 325]
Mean EQ-VAS (SD) [N]	52.5 (21.8) [*N* = 147]	52.8 (20.1) [*N* = 87]	45.6 (20.5) [*N* = 55]	57.6 (21.3) [*N* = 36]	51.9 (21.2) [*N* = 325]
Mean current C19-YRS overall health^d^ (SD) [N]	4.7 (1.9) [*N* = 70]	4.8 (1.8) [*N* = 35]	4.8 (1.8) [*N *= 13]	4.8 (2.0) [*N* = 29]	4.7 (1.9) [*N* = 147]
Mean current C19-YRSm overall health^d^ (SD) [N]	4.4 (2.0) [*N* = 99]	4.5 (1.9) [*N* = 62]	4.3 (2.0) [*N* = 44]	5.5 (2.0) [*N* = 6]	4.4 (2.0) [*N* = 211]
Mean current C19-YRS symptom severity^e^ (SD) [N]	41.2 (18.7) [*N* = 70]	38.2 (17.6) [*N* = 35]	44.8 (15.8) [*N* = 13]	40.5 (16.4) [*N* = 29]	40.7 (17.7) [*N* = 147]
Mean current C19-YRSm symptom severity^e^ (SD) [N]	18.2 (5.8) [*N* = 99]	18.4 (6.1) [*N* = 62]	19.0 (5.8) [*N* = 44]	13.3 (7.5) [*N* = 6]	18.3 (5.9) [N = 211]
Mean current C19-YRS functional disability^f^ (SD) [N]	18.6 (10.5) [*N* = 70]	15.6 (11.4) [*N* = 35]	19.6 (11.4) [*N* = 13]	17.6 (11.3) [*N* = 29]	17.8 (10.9) [*N* = 147]
Mean current C19-YRSm functional disability^f^ (SD) [N]	7.6 (3.5) [*N *= 99]	7.6 (3.9) [*N* = 62]	7.8 (4.0) [*N* = 44]	4.2 (3.4) [*N* = 6]	7.6 (3.8) [*N* = 211]
Mean C19-YRS other symptoms^g^ (SD) [N]	18.9 (12.4) [*N* = 70]	15.5 (10.7) [*N* = 35]	18.4 (10.2) [*N* = 13]	13.8 (12.8) [*N* = 29]	17.0 (12.0) [*N *= 147]
Mean C19-YRSm other symptoms^g^ (SD) [N]	5.9 (3.7) [*N* = 99]	6.5 (4.5) [*N* = 62]	7.7 (4.9) [*N* = 44]	4.2 (4.4) [*N* = 6]	6.4 (4.3) [*N* = 211]
Any secondary care use in previous month N (%)	41 (25.2)	17 (16.8)	15 (24.6)	11 (26.8)	84 (23.0)
Any primary or social care use in previous month N (%)	64 (39.3)	29 (28.7)	19 (31.1)	15 (36.6)	127 (34.7)
Mean number of services received at initial contact with LC clinic (SD)^h^	1.5 (1.1)	1.6 (1.1)	1.8 (1.3)	1.4 (1.1)	1.6 (1.2)
Service received at initial contact with LC clinic *N* (%)^h^					
hone/online consult	97 (59.5)	61 (60.4)	35 (57.4)	19 (46.3)	212 (57.9)
Face-to-face consult	59 (36.2)	34 (33.7)	20 (32.8)	16 (39.0)	129 (35.2)
PT	13 (8.0)	16 (15.8)	15 (24.6)	4 (9.8)	48 (13.1)
OT	22 (13.5)	13 (12.9)	8 (13.1)	5 (12.2)	48 (13.1)
SLT	2 (1.2)	1 (1.0)	1 (1.6)	0 (0.0)	4 (1.1)
Fatigue management	15 (9.2)	4 (4.0)	5 (8.2)	7 (17.1)	31 (8.5)
Counselling	7 (4.3)	7 (6.9)	4 (6.6)	2 (4.9)	20 (5.5)
Peer support	2 (1.2)	1 (1.0)	5 (8.2)	1 (2.4)	9 (2.5)
Dietitian	6 (3.7)	1 (1.0)	4 (6.6)	1 (2.4)	12 (3.3)
Welfare advice	6 (3.7)	0 (0.0)	2 (3.3)	1 (2.4)	9 (2.5)
Multidisciplinary	2 (1.2)	5 (5.0)	5 (8.2)	1 (2.4)	13 (3.6)
Other	15 (9.2)	11 (10.9)	4 (6.6)	2 (4.9)	32 (8.7)

^a^This category combined 2007 Standard Industry Classification (SIC) categories ‘Financial and insurance activities’ and ‘Information and communication’

^b^This category combined 2007 SIC categories ‘Accommodation and food service activities’, ‘Transportation and storage’, and ‘Wholesale and retail trade’

^c^This category combined 2007 SIC categories ‘Arts, entertainment, and recreation’ and ‘Other industries’ and student

^d^Current as in the survey date. Score range for overall health is 0–10 for both original and modified versions of C19-YRS; higher score implies better overall health

^e^Current as in the survey date. Score ranges for symptom severity subscale are 0–100 and 0–30 for C19-YRS original and modified versions, respectively; higher score implies greater severity

^f^Current as in the survey date. Score ranges for functional disability subscale are 0–50 and 0–15 for C19-YRS original and modified versions, respectively; higher score implies greater severity

^g^Score ranges for other symptoms subscale are 0–60 and 0–25 for C19-YRS original and modified versions, respectively; higher score implies greater severity

^h^There were eight missing responses for the type and number of services received at initial contact with LC clinic. These are not disaggregated by duration of LC

*ICT* information and communication technology, *IMD* index of multiple deprivation; *LC* Long COVID, *N/A*: not applicable; *OT* occupational therapy; *PT* physiotherapy; *SD* standard deviation; *SIC* UK Standard Industry Classification code, *SLT* speech and language therapy

### Impact on productivity

**Fig. 1:**
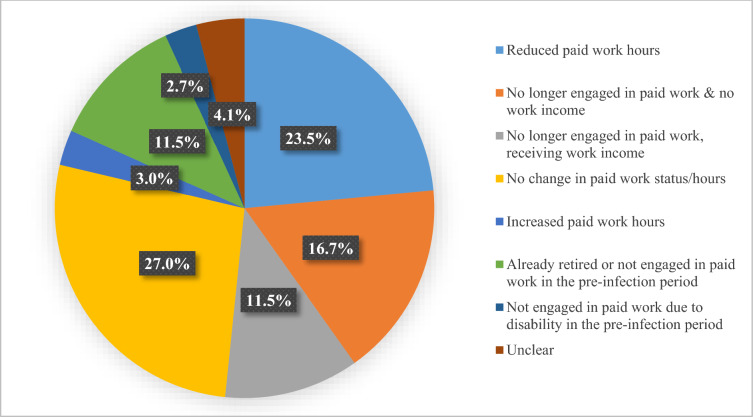
Change in paid work status/h from the pre-infection period to the previous month

Figure 1 displays the proportions of participants by the change in their paid work status/h from the pre-infection period to the previous month. Over half (189 of 366; 51.7%) of the sample had reduced paid work hours or no longer engaged in paid work with or without income. The proportion that returned to pre-infection paid work status/h or increased their paid work hours was 30.0% (110 of 366). Figures A1–A3 in the Supplementary Material display the respective proportions by duration of LC.

Data on the change in unpaid work status/h was available from 53 participants. Of these, 13 (24.5%) reduced unpaid work hours, 26 (49.1%) stopped unpaid work, 7 (13.2%) maintained the same hours as the pre-infection period, and another 7 (13.2%) increased their work hours.

**Fig. 2:**
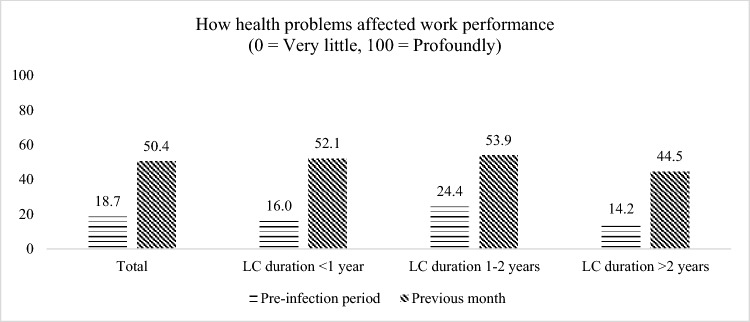
Change in health impact on work performance. *LC* Long COVID

Figure 2 shows the average work performance by duration of LC. All subgroups experienced marked worsening in work performance from the pre-infection period to the previous month.

**Fig. 3:**
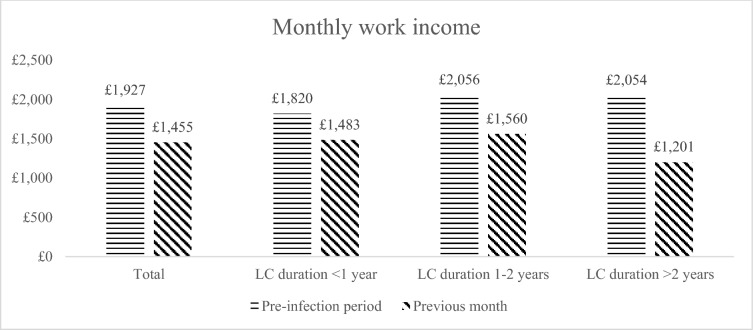
Change in monthly work income, *LC* Long COVID

Figure 3 similarly summarises average monthly work income by duration of LC. In proportional terms, monthly income declined by 24.5% for the whole sample and by 18.5%, 24.1%, and 41.5% for the three respective subgroups defined by duration of LC.

Table 3 shows the bootstrapped mean monetary values of the aggregate and monthly productivity losses from paid work hour reduction by sex, age group, ethnicity, IMD quintile, duration of LC, and industry group. Due primarily to their longer disease duration, those with LC duration longer than 2 years incurred the largest aggregate loss relative to those with LC for less than 2 years. Those working in the health and education sectors incurred larger aggregate and monthly losses (£13,864 and £1,000) relative to those working in financial, ICT, and professional services (£10,300 and £792) and in other industries (£9,341 and £761).

**Table 3:** Mean aggregate and monthly productivity losses from paid work hour reduction^a^

Subgroup	Bootstrap mean^b^ [SE] (95% confidence interval), £			
	Engaged in paid work pre-infection		Whole sample	
	Aggregate value^c^	Monthly value	Aggregate value^c^	Monthly value
All	12,897 [1214] (10,518–15,277)	897 [60] (779–1016)	10,929 [1064] (8844 – 13,014)	763 [54] (657–869)
Sex				
Male	17,232 [2686] (11,968 – 22,496)	1128 [135] (864–1,393)	14,278 [2339] (9694–18,862)	940 [119] (707 – 1173)
Female	10,730 [1192] (8392 – 13,067)	788 [63] (665–911)	9,197 [1056] (7128–11,266)	677 [58] (564–790)
Age group				
< 35 years	13,954 [3439] (7214 – 20,693)	963 [165] (640 – 1,287)	11,231 [2877] (5591 – 16,870)	796 [145] (511 – 1,080)
35–44 years	14,070 [2661] (8853 – 19,285)	967 [115] (742 – 1195)	13,643 [2582] (8582 – 18,704)	944 [113] (722 – 1166)
45–54 years	9,801 [1547] (6770 – 12,833)	797 [109] (584 – 1,010)	9001 [1446] (6,167 – 11,836)	719 [100] (523–915)
≥ 55 years	15,178 [2610] (10,062 – 20,294)	926 [113] (704 – 1,147)	10,905 [1973] (7,038 – 14,771)	667 [89] (493–842)
Ethnicity				
White	13,240 [1407] (10,483 – 15,997)	928 [73] (784 – 1,072)	11,171 [1214] (8791 – 13,551)	793 [65] (666 – 921)
Minority ethnic	11,474 [3550] (4517 – 18,431)	720 [169] (388 – 1051)	9835 [3078] (3802 – 15,868)	620 [150] (326 – 913)
IMD quintile				
Most deprived	15,051 [2877] (9412 – 20,690)	960 [138] (689 – 1,231)	12,901 [2636] (7735 – 18,067)	823 [131] (566–1080)
2nd	11,077 [2216] (6734 – 15,419)	966 [182] (610 – 1,322)	9580 [2012] (5637 – 13,523)	813 [165] (490–1137)
3rd	8,980 [3443] (2232 – 15,729)	833 [264] (316 – 1,350)	7395 [2901] (1710 – 13,081)	686 [227] (241–1,131)
4th	6,634 [1777] (3151 – 10,116)	611 [130] (357 – 866)	5578 [1540] (2560 – 8,596)	518 [114] (296–741)
Least deprived	14,441 [3316] (7943 – 20,940)	1,073 [182] (717 – 1,430)	11,983 [2859] (6381 – 17,586)	894 [160] (580–1209)
LC duration				
< 1 year	7385 [675] (6061 – 8708)	1004 [94] (818–1189)	6012 [594] (4848–7176)	817 [82] (656 – 978)
1–2 years	12,878 [1952] (9053 – 16,704)	785 [112] (566–1,005)	11,658 [1808] (8114–15,202)	711 [104] (507 – 915)
> 2 years	27,517 [4811] (18,088–36,946)	902 [157] (594–1210)	23,172 [4202] (14,936 –31,409)	759 [137] (490 – 1029)
Industry group				
Health and education	13,864 [2,099] (9,751 – 17,978)	1000 [117] (771 – 1229)	N/A	N/A
Financial, ICT and professional	10,300 [2,662] (5081 – 15,518)	792 [153] (493 – 1091)	N/A	N/A
Other industries	9341 [1,711] (5988–12,695)	761 [105] (555 – 967)	N/A	N/A

^a^Positive value implies productivity *loss* from paid work hour reduction

^b^Nonparametric bootstrapping with replacement involved 1,000 replications

^c^Aggregate value is the monthly value of productivity loss multiplied by the number of months since the incident COVID-19 infection that resulted in LC

*ICT* information and communication technology, *IMD* index of multiple deprivation, *LC* Long COVID, *N/A* not applicable, *SE* standard error

Extrapolating from the mean values by LC duration (whole sample), the national aggregate productivity loss due to LC for those whose LC symptoms affected their daily activities ‘a lot’ amounted to £5.7 billion (95% CI: £3.8 to £7.6 billion) and the monthly loss to £277.7 million (95% CI: £196.3 to £359.2 million).

### Impact on informal care receipt

Table 4 shows the pattern of informal care support received by participants by duration of LC. Around a third (31.7%) of participants received some informal care. Most (58.8%) of the primary caregivers were partners or spouses of participants. Almost a quarter (22.8%) of caregivers were reported as having reduced paid or unpaid work hours to accommodate their caregiving.

**Table 4:** Receipt of informal care by duration of Long COVID

	LC duration < 1 year (*N* = 161)	LC duration 1–2 years (*N* = 97)	LC duration > 2 years (*N* = 61)	LC duration unknown (*N* = 41)	Total (*N* = 360)
Receiving any informal care N (%)	58 (36.0)	24 (24.7)	20 (32.8)	12 (29.3)	114 (31.7)
Primary caregiver^a^ relation *N* (% of care recipients)					
Partner/spouse	41 (70.7)	11 (45.8)	12 (60.0)	3 (25.0)	67 (58.8)
Sibling	1 (1.7)	2 (8.3)	0 (0.0)	1 (8.3)	4 (3.5)
Child	7 (12.1)	6 (25.0)	5 (25.0)	4 (33.3)	22 (19.3)
Parent/grandparent	6 (10.3)	1 (4.2)	2 (10.0)	1 (8.3)	10 (8.8)
Friend/colleague	3 (5.2)	1 (4.2)	0 (0.0)	2 (16.7)	6 (5.3)
Missing	0 (0.0)	3 (12.5)	1 (5.0)	1 (8.3)	5 (4.4)
Mean weekly care hours^b^ (SD)	19.7 (19.8)	23.7 (27.3)	21.5 (13.4)	27.0 (17.5)	21.3 (19.9)
Median weekly care hours^b^ (IQR)	12.0 (7.0–28.0)	14.0 (5.0–33.0)	20.0 (12–26.5)	29.0 (10.0–40.0)	15.0 (8.0–28.0)
Caregiver(s) reduced work hours N (% of care recipients)	11 (19.0)	9 (37.5)	5 (25.0)	1 (8.3)	26 (22.8)

^a^Participants listed up to three caregivers. Only the primary caregivers’ relations are presented here

^b^Includes care hours provided by up to three caregivers

*IQR* interquartile range; *LC* long COVID; *SD* standard deviation

Table 5 shows the bootstrapped mean aggregate and monthly monetary values of informal care receipt by sex, age group, ethnicity, IMD quintile, and duration of LC. Like productivity loss, the aggregate value of informal care received was greater for those who spent more than 2 years with LC relative to those who spent less, owing primarily to their LC duration.

**Table 5:** Mean aggregate and monthly value of informal care receipt

Subgroup	Bootstrap mean^a^ [SE] (95% confidence interval), £			
	Receiving any informal care		Whole sample	
	Aggregate value^b^	Monthly value	Aggregate value^b^	Monthly value
All	28,246 [3,404] (21,574 – 34,917)	1,911 [166] (1,585 – 2237)	8,726 [1,265] (6,247 – 11,204)	583 [69] (448 – 718)
Sex				
Male	34,040 [9957] (14,524 – 53,556)	1657 [268] (1133 – 2182)	5726 [2029] (1751 – 9702)	283 [73] (141 – 426)
Female	26,925 [3322] (20,413 – 33,437)	1,969 [181] (1613 – 2325)	10,276 [1573] (7192–13,360)	731 [90] (555 – 907)
Age group				
< 35 years	16,660 [5441] (5997 – 27,324)	1280 [241] (807 – 1753)	4,922 [1955] (1,091–8,754)	366 [101] (168 – 563)
35–44 years	24,665 [5386] (14,108 – 35,222)	2219 [314] (1604 – 2,834)	9,716 [2532] (4,754–14,679)	797 [158] (487 – 1,106)
45–54 years	30,812 [5914] (19,220 – 42,404)	1845 [247] (1361 – 2,329)	9,457 [2337](4,877–14,037)	573 [114] (350 – 795)
≥ 55 years	34,325 [7559] (19,508 – 49,141)	1997 [366] (1279 – 2715)	8,998 [2390] (4,313 – 13,683)	540 [123] (300 – 780)
Ethnicity				
White	29,398 [3744] (22,060 – 36,736)	1877 [182] (1521 – 2233)	9,385 [1433] (6,576 – 12,193)	592 [76] (443 – 742)
Minority ethnic	18,452 [3066] (12,444 – 24,461)	1965 [338] (1303 – 2628)	6,363 [1927] (2,586 – 10,140)	584 [171] (248 – 920)
IMD quintile				
Most deprived	57,098 [11,284] (34,982 – 79,214)	3201 [515] (2191 – 4,211)	21,246 [5859] (9763 – 32,729)	1,191 [295] (613 – 1,769)
2nd	24,272 [7002] (10,548 – 37,995)	1767 [347] (1088 – 2447)	9184 [3187] (2,938 – 15,430)	698 [191] (323 – 1,073)
3rd	25,196 [9085] (7390 – 43,003)	1321 [297] (739 – 1904)	7411 [3387] (773 – 14,049)	389 [136] (122 – 655)
4th	11,236 [3559] (4260 – 18,212)	962 [167] (634 – 1289)	3420 [1,269] (933 – 5906)	300 [83] (138 – 463)
Least deprived	22,374 [5518] (11,560 – 33,189)	2382 [669] (1071 – 3692)	5817 [1,912] (2,071 – 9,564)	607 [222] (171 – 1,043)
LC duration				
< 1 year	13,417 [1661] (10,161 – 16,674)	1800 [216] (1376 – 2224)	4726 [754] (3247 – 6204)	634 [100](438 – 830)
1–2 years	34,625 [7736] (19,463 – 49,787)	2047 [471] (1, 24 – 2970)	7735 [2207] (3411 – 12060)	457 [130] (202 – 713)
> 2 years	63,066 [8741] (45,935 – 80,197)	1992 [287] (1429 – 2555)	20,677 [4852] (11,168 – 30,187)	653 [156] (348 – 959)

^a^Nonparametric bootstrapping with replacement involved 1,000 replications

^b^Aggregate value is the monthly value of informal care receipt multiplied by the number of months since the incident COVID-19 infection that resulted in LC

*LC* Long COVID, *N/A* not applicable, *SE* standard error

Extrapolating from the mean values by LC duration, the national aggregate informal care value for those whose LC symptoms impacted their daily activities ‘a lot’ amounted to £4.8 billion (95% CI: £2.6 to £7.0 billion) and the monthly value to £218.2 million (95% CI: £122.4 to £314.2 million).

### Multivariate analyses

Table 6 shows the results of multilevel logistic regressions on the likelihood of returning to the same or increased paid work hours as the pre-infection period, adjusted for alternative sets of covariates. In covariate set (4), which included covariates with *P*-values less than 0.10 in set (3), there was a significant positive association between LC duration and return to the same or increased paid work hours. Those who spent 1–2 years with LC had 2.668 times increased odds of returning than those who spent less than one year (*P* = 0.007). The odds ratio was 2.571 (*P* = 0.040) for those who spent longer than 2 years. Those who used any community health or social care service in the previous month were less likely to have returned to the same or increased paid work hours (OR 0.477; *P* = 0.027). A 0.01-unit improvement in the EQ-5D-3L utility score was associated with 1.026 increased odds (*P* = 0.002). Being in the highest quartile for the increase in C19-YRS(m) functional disability was associated with lower odds (OR 0.236; *P* = 0.009). The latter two findings support the a priori hypothesis of significant associations between LC severity as measured by the two PROMs and the productivity impact of LC.

**Table 6:** Multivariate analysis of returning to same/higher paid work hours as the pre-infection period

Model: multilevel logistic regression^a,b^	(1) Demographics [*N* = 270]		(2) COVID-19 + (1) [N = 266]		(3) PROMs + (2) [N = 209]		(4) *P* < 0.1 variables in (3) [N = 250]	
	OR (SE)	*P* value	OR (SE)	*P* value	OR (SE)	*P*-value	OR (SE)	*P*-value
Constant	0.380 (0.331)	0.267	0.815 (0.805)	0.836	1.151 (1.642)	0.921	0.141** (0.085)	0.001
Pre-infection period paid work hours	0.992 (0.014)	0.556	0.987 (0.014)	0.371	0.971 (0.020)	0.159		
LC duration (ref: < 1 year)								
1–2 years	2.164* (0.731)	0.022	1.790 (0.611)	0.088	2.170 (0.993)	0.091	2.668** (0.966)	0.007
> 2 years	1.755 (0.698)	0.157	1.697 (0.709)	0.205	3.234* (1.839)	0.039	2.571* (1.180)	0.040
Missing	1.684 (0.828)	0.290	1.394 (0.723)	0.521	1.919 (1.427)	0.381	1.873 (0.927)	0.205
Male (ref: female)	0.927 (0.301)	0.814	0.959 (0.325)	0.903	0.645 (0.304)	0.353		
Age group (ref: < 35)								
35–44 years	1.180 (0.548)	0.722	1.083 (0.517)	0.867	1.196 (0.759)	0.778		
45–54 years	1.365 (0.598)	0.477	1.189 (0.540)	0.702	1.091 (0.628)	0.880		
55–64 years	1.658 (0.796)	0.292	1.554 (0.767)	0.372	1.125 (0.712)	0.853		
≥ 65 years	0.594 (0.224)	0.580	0.430 (0.422)	0.390	0.242 (0.289)	0.235		
White ethnicity (ref: minority ethnic)	0.517 (0.224)	0.128	0.674 (0.299)	0.373	1.216 (0.767)	0.756		
IMD quintile (ref.: most deprived)								
2nd	1.388 (0.843)	0.589	1.441 (0.925)	0.570	0.683 (0.574)	0.651		
3rd	1.840 (1.081)	0.300	1.581 (0.978)	0.459	0.534 (0.498)	0.501		
4th	3.745* (2.222)	0.026	3.992* (2.454)	0.024	1.229 (1.020)	0.804		
Least deprived	2.242 (1.234)	0.143	2.122 (1.183)	0.177	1.038 (0.825)	0.963		
Missing	1.802 (1.008)	0.292	1.691 (0.930)	0.340	0.780 (0.693)	0.780		
Industry group (ref: health and education)								
Financial, ICT and professional	1.902 (0.756)	0.106	1.799 (0.747)	0.157	1.024 (0.554)	0.965		
Other industries	0.869 (0.335)	0.716	0.816 (0.328)	0.613	0.783 (0.386)	0.620		
Missing	0.921 (0.352)	0.829	0.880 (0.337)	0.739	1.151 (0.653)	0.804		
Hospitalised for COVID-19 (ref: not hospitalised)			1.663 (0.808)	0.295	1.861 (1.350)	0.392		
COVID-19 vaccination (ref: single vaccinated)								
Double-vaccinated			0.705 (0.291)	0.396	0.596 (0.330)	0.350		
Missing			0.998 (0.489)	0.997	0.693 (0.512)	0.620		
Secondary care use in prev. month (ref: no use)			0.667 (0.241)	0.263	0.867 (0.432)	0.774		
Community health/social care use in prev. month (ref: no use)			0.404** (0.126)	0.004	0.327** (0.135)	0.007	0.477* (0.160)	0.027
Number of services received at LC clinic			0.944 (0.122)	0.658	0.988 (0.184)	0.949		
EQ-5D-3L utility rescaled from 0–1 to 0–100					1.023* (0.011)	0.039	1.026** (0.008)	**0.002**
Highest quartile for								
Increase in C19-YRS(m) symptom severity					0.472 (0.264)	0.179		
Increase in C19-YRS(m) functional disability					0.188* (0.128)	0.014	0.236** (0.130)	**0.009**
Improvement in C19-YRS(m) overall health					1.271 (0.591)	0.606		
C19-YRS(m) other symptoms					0.711 (0.390)	0.535		

^a^Participants are grouped by region: Birmingham; Cardiff; Hertfordshire; Leeds; Leicester; London; Newcastle; Oxford; Salford. Complete cases are used. Odds ratios, standard errors, and *P* values are reported to three decimal places

^b^Statistical significance: * *P* < 0.05; ** *P* < 0.01

*ICT* information and communication technology, *IMD* index of multiple deprivation, *LC* Long COVID, *OR* odds ratio, *prev.* previous, *PROM* patient-reported outcome measure, *ref* reference *SE* standard error

Tables A3–A5 in the Supplementary Material show, respectively, the results of multilevel linear regressions on the change in paid work hours relative to the pre-infection period, the change in work performance having returned to paid/unpaid work, and the change in monthly work income, adjusted for each of four alternative sets of covariates. A higher EQ-5D-3L utility score was significantly and positively associated with all three changes as hypothesised, indicating higher positive change (or lesser decline) in the three outcomes. Being in the highest quartile for the improvement (or lesser decline) in C19-YRS(m) overall health was significantly associated with higher work hours and less health issues affecting work performance. By contrast, no C19-YRS(m) subscale was significantly associated with change in monthly income.

Table 7 shows the results of multilevel logistic regressions on the likelihood of receiving any informal care, adjusted as above. Based on covariate set (4), a higher EQ-5D-3L utility score was significantly associated with lower odds of receiving informal care (OR 0.979; *P* < 0.001), while being in the highest quartile for the increase in C19-YRS(m) symptom severity (OR 2.866; *P* = 0.001) was significantly associated with increased odds of receiving informal care. Those who spent more than 1 year with LC were less likely to require informal care, but the association was statistically significant only for those who spent 1–2 years with LC (OR 0.438; *P* = 0.035).

**Table 7:** Multivariate analysis of receiving any informal care

Model: multilevel logistic regression^a,b^	(1) Demographics [*N* = 321]		(2) COVID-19 + (1) [*N* = 315]		(3) PROMs + (2) [*N* = 252]		(4) *P* < 0.1 variables in (3) [N = 301]	
	OR (SE)	*P*-value	OR (SE)	*P*-value	OR (SE)	*P*-value	OR (SE)	*P*-value
Constant	1.067 (0.652)	0.915	0.464 (0.356)	0.317	1.109 (1.216)	0.925	0.851 (0.335)	0.681
LC duration (ref: < 1 year)								
1–2 years	0.634 (0.199)	0.146	0.690 (0.240)	0.287	0.525 (0.240)	0.159	0.438* (0.171)	0.035
> 2 years	0.987 (0.351)	0.972	1.197 (0.458)	0.637	0.582 (0.280)	0.261	0.519 (0.212)	0.108
Missing	0.562 (0.291)	0.265	0.648 (0.352)	0.424	0.199* (0.163)	0.048	0.554 (0.287)	0.254
Male (ref: female)	0.340** (0.106)	0.001	0.320** (0.109)	0.001	0.559 (0.109)	0.148		
Age group (ref: < 35)								
35–44 years	1.773 (0.734)	0.167	1.935 (0.852)	0.134	1.235 (0.682)	0.702		
45–54 years	1.250 (0.495)	0.572	1.428 (0.597)	0.394	0.759 (0.385)	0.586		
55–64 years	1.235 (0.530)	0.622	1.246 (0.569)	0.630	0.602 (0.328)	0.352		
≥ 65 years	1.207 (0.790)	0.773	1.483 (1.015)	0.565	1.234 (1.007)	0.797		
White ethnicity (ref: minority ethnic)	0.878 (0.359)	0.750	0.752 (0.336)	0.524	0.935 (0.604)	0.917		
IMD quintile (ref.: most deprived)								
2nd	0.847 (0.428)	0.743	1.072 (0.603)	0.902	1.080 (0.786)	0.916		
3rd	0.704 (0.360)	0.493	0.910 (0.508)	0.866	1.879 (1.401)	0.397		
4th	0.603 (0.306)	0.319	0.832 (0.450)	0.733	1.958 (1.345)	0.328		
Least deprived	0.504 (0.239)	0.149	0.679 (0.346)	0.448	1.131 (0.768)	0.857		
Missing	0.517 (0.210)	0.105	0.566 (0.289)	0.266	1.124 (0.775)	0.866		
Industry group (ref: health and education)								
Financial, ICT and professional	1.080 (0.440)	0.850	1.337 (0.590)	0.510	1.690 (0.886)	0.317		
Other industries	0.748 (0.289)	0.452	0.928 (0.382)	0.855	0.722 (0.361)	0.515		
Missing	1.090 (0.344)	0.784	1.344 (0.461)	0.389	0.976 (0.459)	0.960		
Hospitalised for COVID-19 (ref: not hospitalised)			2.145 (0.961)	0.088	2.859 (1.739)	0.084	2.021 (1.004)	0.157
COVID-19 vaccination (ref: single vaccinated)								
Double-vaccinated			1.086 (0.435)	0.837	0.977 (0.478)	0.961		
Missing			0.993 (0.498)	0.989	1.184 (0.744)	0.788		
Secondary care use in prev. month (ref: no use)			1.716 (0.551)	0.093	1.202 (0.499)	0.657		
Community health/social care use in prev. month (ref: no use)			3.065^¶^** (0.856)**	< 0.001	2.222* (0.782)	0.023	2.287** (0.669)	0.005
Number of services received at LC clinic			0.889 (0.113)	0.353	0.787 (0.130)	0.147		
EQ-5D-3L utility rescaled from 0–1 to 0–100					0.979* (0.008)	0.012	0.979^¶^ (0.005)	< 0.001
Highest quartile for								
Increase in C19-YRS(m) symptom severity					3.090** (1.291)	**0.007**	2.866** (0.944)	0.001
Increase in C19-YRS(m) functional disability					1.409 (0.671)	0.471		
Improvement in C19-YRS(m) overall health					0.833 (0.386)	0.693		
C19-YRS(m) other symptoms					1.294 (0.553)	0.546		

^a^Participants are grouped by region: Birmingham; Cardiff; Hertfordshire; Leeds; Leicester; London; Newcastle; Oxford; Salford. Complete cases are used. Odds ratios, standard errors, and *P* values are reported to three decimal places

^b^Statistical significance: * *P* < 0.05; ** *P* < 0.01; *P* < 0.001

*ICT* information and communication technology, *IMD* index of multiple deprivation, *LC* Long COVID, *OR* odds ratio; *prev.* previous, *PROM* patient-reported outcome measure, *ref* reference, *SE* standard error

The EQ-5D-3L utility score was included as an explanatory variable in covariate set (4) in each of Tables 6, 7 and A3-A5. Accordingly, Tables A6–A10 in the Supplementary Material show the results of multivariate regressions for the same dependent variables where the EQ-5D-3L utility score has been replaced by individual EQ-5D-5L dimension responses as covariates. The EQ-5D-5L dimensions differed in their associations with the dependent variables. For example, the odds of returning to same or increased paid work hours (Table A6) was significantly and negatively associated with having moderate problems (relative to having no problems) for mobility (OR 0.201; *P* < 0.001), self-care (OR 0.181; *P* = 0.012), and usual activities (OR 0.361; *P* = 0.042), but not for pain/discomfort (OR 0.678; *P* = 0.396) and anxiety/depression (OR 0.563; *P* = 0.178).

The multivariate analyses found no evidence of variation in productivity losses and informal care receipt by key sociodemographic variables, including sex, ethnicity, and IMD quintile, though there was some evidence that individuals working in the financial, ICT, and professional sectors were better shielded from paid work hour and monthly work income reductions than those working in the health and education sectors (Tables A3 and A5, respectively, and see “Discussion”).

## Discussion

This study estimated and valued productivity losses and informal care receipt within a cross-sectional sample of LC patients referred to specialist LC care clinics in the UK. These economic impacts were found to be substantial, with 23.5% of the sample reducing their paid work status/h, 28.2% no longer engaging in paid work, the average monthly income falling by 24.5%, and 31.7% of the sample requiring informal care. The monetary value of the productivity loss since the incident infection amounted to around £277.7 million per month and £5.7 billion in aggregate amongst those whose LC symptoms impacted their daily activities ‘a lot’, when extrapolated nationally across the UK. The respective estimates for the value of informal care were £218.2 million and £4.8 billion. As hypothesised, the EQ-5D-3L utility score and at least one C19-YRS(m) subscale were shown to be significantly associated with all productivity and informal care outcomes, except for change in monthly income, assessed in multivariate analyses.

The substantial productivity loss associated with LC estimated by this study is broadly consistent with those of previous UK-based studies. Walker and colleagues [10] found that among LC patients referred to LC specialist clinics in the UK (n = 3,754), 50.8% had lost one or more working days in the previous month and 20.3% lost between 20 and 28 working days. Ziauddeen and colleagues [28] found that 19.1% of an online sample of LC patients in the UK (n = 2,550) reported being unable to work and 66.4% taking time off sick. In comparison, 51.7% in this sample had reduced work hours or stopped employment. Reuschke and Houston [11] estimated that around 80,000 persons in the UK whose daily activities had been affected ‘a lot’ by LC had left employment by March 2022. Extrapolating nationally using the same ONS data source [2] as Reuschke and Houston, this study estimates that 102,648 LC patients of similar disease severity had left employment by March 2023.

The substantial productivity impact of LC is clear when the labour market activity of LC patients is compared to that of the general UK population. According to the UK Department for Work & Pensions [65], the proportion economically inactive or unemployed in 2022 was 14.5% for those aged 35–49 years (9.4% for men, 19.4% for women) and 30.0% for those aged 50–64 years (26.0%, 33.9%). The corresponding proportions in our study sample were 36.8% for those aged 35–49 years (38.7%, 36.3%) and 43.2% for those aged 50–64 years (31.7%, 51.1%). Proportionally, therefore, the productivity impact has been most acute for male LC patients aged 35–49 years whose rate of economic inactivity or unemployment is 411.7% of that of their UK general population peers [65]. It should be noted that the highest rate of LC incidence in the UK has been found among the older working age population aged 45–69 years [66]. This study hence finds that the younger working age population has been disproportionately affected in terms of labour market participation.

The high volume of non-UK (mainly European) research on the productivity impacts of LC [9, 12–20, 22–26] allows international comparison of the impacts, though a comprehensive synthesis is beyond the scope of this paper. A multinational cohort study of 3,762 LC patients found that 45.2% of patients had reduced work hours and 22.3% no longer engaged in paid work [9]. In comparison, the current study sample comprised a higher proportion (28.2%) of those dropping out of the labour market but a much lower proportion (23.5%) returning to work at reduced schedules. A Swiss study of a sample containing 120 LC patients found 1.6% dropping out of employment and 5.8% having work ability being affected by LC [12]. Relative to the UK studies, this represents a much lower proportion of LC patients whose productivity has been adversely affected. The difference could primarily be attributed to the lower disease severity in the Swiss sample with only 6.7% of the patients having severe health impairment [12]. It is nevertheless feasible that between-country variation in occupational support structures have played a role [67]. A mixed-methods study in the UK, for example, identified key enablers to work return such as line management competency [21], and further research is warranted on whether and to what extent such environmental factors explain the between-country and between-sector differences in productivity outcomes.

Further comparisons can be made to other serious chronic diseases. The UK employment rates for patients with myalgic encephalomyelitis/chronic fatigue syndrome (ME/CFS) [68], multiple sclerosis [68], and non-metastatic breast cancer [69] have been estimated to be 35%, 60%, and 50.9% (women only), respectively. The rate for the current sample was 53.5% (52.8% women only), suggesting that the employment loss from LC is similar to those from multiple sclerosis and non-metastatic breast cancer, though less severe than that from ME/CFS. That said, the higher prevalence of LC relative to ME/CFS means that the total productivity loss from LC is likely much greater. A previous study estimated the annual UK-wide productivity cost due to discontinued employment after ME/CFS onset to be £102.2 million in 2009 prices (£143.6 million in 2022 prices) [70]. This compares to the annual national cost of £3.3 billion (95% CI £2.4 to £4.3 billion) when the current sample’s monthly estimate is extrapolated, although this figure includes the value of all paid work hour reductions, not only of discontinued employment. The annual cost of productivity loss from incident cases of early breast cancer has been estimated at £141.4 million (£168.5 million in 2022 prices) [71]. Overall, the productivity impact of LC can be seen to be as significant as those of other major chronic diseases.

As hypothesised, the multivariate analyses found significant associations between the EQ-5D-3L utility score derived from EQ-5D-5L dimension responses and all productivity-related dependent variables and receipt of any informal care. By contrast, not all EQ-5D-5L dimension responses were significantly associated with the dependent variables, and it may be feasible to infer from the significant associations which of the dimensions are most strongly related with vocational difficulties and care needs. Mobility, for instance, was significantly associated with all dependent variables, and this may motivate increased focus on mobility rehabilitation. Another key finding was the consistent association between one or more C19-YRS(m) subscale score and dependent variables after adjusting for the EQ-5D-3L utility score. The C19-YRS(m) symptom severity subscale, for example, was independently associated with receipt of any informal care. This suggests that better care strategies and professional support tailored to LC-specific symptoms may significantly alleviate the informal caregiver burden. It also suggests that the generic EQ-5D-5L and disease-specific C19-YRS(m) outcome measures should be used in tandem in clinical practice and research.

There was some evidence from the multivariate analyses that the longer the time since the incident infection, the higher the likelihood of returning to the pre-infection work status/h and the lower the likelihood of receiving any informal care, after adjusting for one’s health status measured by the EQ-5D-3L utility score, community health or social care service use in the previous month, and C19-YRS(m) symptom severity or functional disability. However, given that those with LC duration longer than 2 years (the ‘long-haulers’) have significantly worse health status than those with shorter durations as measured by EQ-5D-3L utility score (difference of -0.115 compared to those with LC duration less than 2 years; *t* test *P* value of 0.0078), the finding suggests that the long-haulers are returning to work and/or managing without care support *despite* their worse health status. Financial pressure could be a reason for the work return, as could a desire to limit the care burden on families. More detailed research on this subset of LC long haulers is warranted, particularly regarding their employment, financial, and care support situations.

The substantial magnitude of productivity loss and informal caregiver burden in this patient sample strongly suggest that economic evaluations of LC intervention strategies conducted from a societal perspective should include these outcomes. Such an approach has been previously recommended for ME/CFS, a disease which in some respects may be similar in aetiology and symptoms to LC [72], as well as for a broader family of complex public health interventions [49, 73, 74]. The prevalence data on productivity change and informal care receipt from this study, as well as their final valued outcomes, should serve as important inputs into model-based economic evaluations of LC interventions. Nevertheless, it is likely that equity concerns are particularly relevant for economic evaluations conducted from a societal perspective: the disadvantage of those with the most severe impairments and/or from socially deprived or marginalised backgrounds may be further highlighted when outcomes such as productivity changes are considered in addition to health [75].

Considering such equity concerns, it is noteworthy that the multivariate analyses failed to find any significant evidence of variation in productivity loss and informal care receipt by indicators of social vulnerability, including ethnicity (white vs. minority ethnic) and IMD quintile. A likely issue is the unrepresentativeness of the study sample (comprising LC patients who are diagnosed and referred to LC specialist clinics) to the general LC patient population in the UK in terms of its ethnic and socioeconomic mix. A larger UK study that used primary care records found that Black, mixed ethnicity and other ethnic minority groups comprised around 7.0% of the patients infected with SARS-CoV-2 between January 2020 and April 2021 and had between 6 and 21% increased risks of developing LC [76]. By contrast, these minority ethnic groups comprised just 4.7% of the current study sample, suggesting that the minority ethnic LC patients are underrepresented as was the case in previous UK COVID-19 trials [77]. Analyses of ethnically unrepresentative samples, including multivariate analyses and national extrapolations of productivity and caregiving values, may produce misleading results [78]. There was no statistically significant difference between the respondents and non-respondents to the HEQ in terms of their ethnicity and IMD quintile. This suggests that improved representation of marginalised groups requires broadening the target population to those who are not yet referred to LC specialist clinics and/or have LC symptoms yet no LC diagnosis. Defining diverse ethnic groups, rather than treating them as a homogenous population, would also avoid masking inequities that affect specific groups.

This study has several further limitations. First, it did not use one of established productivity questionnaires such as the Work Productivity and Activity Impairment questionnaire (WPAI), which would have allowed comparisons with economic estimates for other diseases [79–81]. That said, the study questionnaire was designed with extensive PPI input from LC patients, including discussions on existing questionnaires such as the WPAI. Second, it was unclear how the survey respondents accounted for inflation in reporting their work incomes in the pre-infection period and previous month. This issue has been encountered in a previous study [68], which noted that if inflation was factored in, the income loss would be even greater in real terms. Third, the study did not explore alternative ways of valuing productivity and informal care [42, 47]. For productivity, an alternative method is friction costing [47], which limits the productivity cost to that related to the time to hire and train a replacement worker. In the absence of accurate estimates of this friction cost, a heuristic method might have been to apply a deflator to the annual income used under the human capital approach [82]. For caregiving, the valuation should ideally account also for the health and wellbeing impact on the caregivers [42]. Finally, the cross-sectional study design meant that the direction of association in the multivariate analyses could not be assessed. The EQ-5D-5L anxiety/depression dimension, for example, was significantly associated with higher reduction in monthly work income (Table A8), but it was unclear whether anxiety impaired income-earning or the lower income resulted in greater anxiety (or both). It was likewise not feasible to estimate any impact that LC specialist services might have had on the various outcomes.

Some caveats to the productivity loss valuation should also be noted. First, the values only incorporate losses from paid work hour reductions. They hence exclude the value of unpaid work hour reduction and of poorer work performance due to LC symptoms (i.e. presenteeism). They also exclude the value of informal caregivers’ work reductions. Second, the change in paid work status/h from the pre-infection period to the month preceding the survey may not strictly be due to LC symptoms; external firm-level and macroeconomic factors (e.g. lower economic activity in lockdowns inducing layoffs) may be at play. Third, it was assumed that the extent of paid work status/h change from the pre-infection period to the previous month was constant to calculate the aggregate loss value. The same assumption was made to calculate the aggregate informal care value. If, however, the paid work status at the survey date represents an improvement relative to earlier stages of LC, then the aggregate value may represent an underestimate, and if a decline an overestimate. Further prospective analyses based on longitudinal cohort studies are warranted to understand the individual trajectories in work status/h and care burden from the incident infection and LC diagnosis. Fourth, the loss values were extrapolated only to those whose LC symptoms limited their daily activities ‘a lot’. In addition to the 381,000 individuals in this group, there were 1,075,000 in the UK who reported LC symptoms limiting their daily activities ‘a little’ (323,000 with LC duration < 1 year, 307,000 1–2 years, and 445,000 > 2 years) [2]. Those with unknown LC duration were also excluded from the extrapolation. Accounting for these groups’ losses would likely enlarge the aggregate burden.

Further research is warranted to analyse the qualitative data obtained from the free-text responses (available from 187 of 366 participants; 51.1%) regarding further personal and environmental factors influencing the productivity of LC patients which are only imperfectly captured by the quantitative data. Box A1 in the Supplementary Material presents a selected sample of quotes organised under the themes ‘Pressure to maintain work performance’, ‘Concern over career prospects’, and ‘Financial pressure even in employment’. Finally, further research can use the full set of information in the HEQ to measure and value the disease impact on health and social care utilisation and patient out-of-pocket expenditures. This will enable a comprehensive evaluation of the economic impact of LC delineated by sectors as done previously for similar diseases [82–84].

## Conclusion

LC has major impacts on productivity and informal care needs. The high national prevalence of LC likely implies substantial monetary values of over £5.7 billion from productivity losses and £4.8 billion from informal caregiving costs. Patient-reported outcome measures, including outputs from the generic EQ-5D-5L health-related quality of life measure and condition-specific C19-YRS(m) measure, are significantly associated with productivity- and care-related outcomes, such that the measures can be used for rehabilitation planning and evaluation. Our economic values for productivity changes and informal care receipt can inform model-based economic evaluations of LC interventions. The cross-sectional design is a limitation of this study, and a key future research direction is to understand the longitudinal trajectories of productivity patterns and informal care needs of LC patients. This would facilitate causal inferences on how the symptoms of LC affect these outcomes and enable more accurate estimation and extrapolation of their monetary values. Mixed-methods research identifying the environmental determinants of productivity and care needs would help inform the design of vocational rehabilitation and labour regulation suited to LC patients. Further research is also needed with a more representative sample of people with LC, including those not yet referred to LC specialist clinics and those with LC symptoms but no diagnosis, to identify whether inequalities in productivity loss and informal care receipt affect marginalised groups such as deprived and specific minority ethnic populations.

## Supplementary Information

Below is the link to the electronic supplementary material.

Supplementary file1 (DOCX 143 KB)

## Abbreviations

C19-YRS: COVID-19 Yorkshire Rehabilitation Scale
C19-YRSm: COVID-19 Yorkshire Rehabilitation Scale modified
CI: Confidence interval
DPROM: Digital patient-reported outcome measure
HEQ: Health economics questionnaire
ICT: Information and communication technology
ICU: Intensive care unit
IMD: Index of multiple deprivation
LC: Long COVID
ONS: Office for National Statistics
SE: Standard error
SIC: Standard industry classification
WPAI: Work Productivity and Activity Impairment questionnaire
