# Supplementary material for: Impact of Long COVID on productivity and informal caregiving
Source: Eur J Health Econ. 2023 Dec 26;25(7):1095–115. doi: 10.1007/s10198-023-01653-z (PMC11377524; doi:10.1007/s10198-023-01653-z)
Supplement: Supplementary file 1 — Supplementary file1 (DOCX 143 KB) [file 10198_2023_1653_MOESM1_ESM.docx]

# Impact of Long COVID on productivity and informal caregiving

**Supplementary Material**

# Health Economics Questionnaire

**Initial Long Covid services**

**(1) Please list the services you received at your first contact with a Long Covid clinic (circle all appropriate):**

1. Phone or online consultation
2. Medical doctor consultation
3. Physiotherapy (one-to-one)
4. Occupational therapy (one-to-one)
5. Speech and language therapy (one-to-one)
6. Fatigue management
7. Counselling
8. Peer support group:
9. Dietitian
10. Welfare advice
11. Multidisciplinary group intervention
12. Other, please specify: _______________________________

**(2) Did you or your family incur any private expenses related to your first contact with the Long Covid clinic (e.g., travel costs)?**

1. Yes
2. No

**If ‘Yes’, please list your private expenses:**

| **Expense item** | **Please provide detail:** | **Cost to you or your family** |
| --- | --- | --- |
| Travel to and from the Long Covid clinic |  | £ |
| Prescriptions issued by the Long Covid clinic |  | £ |
| Equipment issued by the Long Covid clinic |  | £ |
| Other expenses: |  | £ |

**Health and social care use**

**(B1) Over the past month, have you had any contact with hospital services not requiring overnight stays for Covid symptoms?**

1. Yes
2. No

**If ‘Yes’, please list the hospital services not requiring overnight stays:**

| **Service type** | **Number of contacts:** | |
| --- | --- | --- |
|  | **Face-to-face** | **Remotely (e.g., online, telephone)** |
| *EXAMPLE: Cardiology outpatient service* | *2* | *0* |
| A&E attendance |  |  |
| Cardiology outpatient service |  |  |
| Neurology outpatient service |  |  |
| Respiratory outpatient service |  |  |
| Haematology outpatient service |  |  |
| Ophthalmology outpatient service |  |  |
| Gastroenterology outpatient service |  |  |
| Psychiatry outpatient service |  |  |
| Physiotherapy |  |  |
| Occupational therapy |  |  |
| Speech and language therapy |  |  |
| Other outpatient services, please specify below: | | |
| 1. |  |  |
| 2. |  |  |
| 3. |  |  |

**(B2) Over the past month, have you had any inpatient hospital admissions requiring overnight stays for Covid symptoms?**

1. Yes
2. No

**If ‘Yes’, please list the hospital inpatient admissions requiring overnight stays:**

| **Admission type** | **Number of admissions:** | **Total number of nights in hospital:** |
| --- | --- | --- |
| *EXAMPLE: Intensive care* | *1* | *3* |
| Intensive care |  |  |
| General ward |  |  |
| Other inpatient services (e.g., cardiac unit, psychiatric ward), please specify below: | | |
| 1. |  |  |
| 2. |  |  |

**(B3) Over the past month, have you had any contacts with community healthcare services (e.g., GP appointments) for Covid symptoms?**

1. Yes
2. No

**If ‘Yes’, please list the community healthcare services:**

| **Service type** | **Number of contacts:** | |
| --- | --- | --- |
|  | **Face-to-face** | **Remotely (e.g., online, telephone)** |
| *EXAMPLE: GP appointment* | *1* | *2* |
| GP appointment |  |  |
| NHS 111 |  |  |
| NHS walk-in centre |  |  |
| GP nurse appointment |  |  |
| District nurse appointment |  |  |
| Ambulance callout (but not taken to hospital) |  |  |
| Mental health nurse appointment |  |  |
| Community psychiatrist appointment |  |  |
| Health visitor appointment |  |  |
| Residential care stay funded by NHS | Number of days: | |
| Other community services, please specify below: | | |
| 1. |  |  |
| 2. |  |  |

**(B4) Over the past month, have you had any contacts with social care services (funded by the local council) for Covid symptoms?**

1. Yes
2. No

**If ‘Yes’, please list the social care services:**

| **Service type** | **Number of contacts:** | |
| --- | --- | --- |
|  | **Face-to-face** | **Remotely (e.g., online, telephone)** |
| Care manager appointment |  |  |
| Social worker appointment |  |  |
| Home care worker appointment |  |  |
| Food, laundry and equipment service |  |  |
| Residential care stay funded by Council | Number of days: | |
| Other social care services, please specify below: | | |
| 1. |  |  |
| 2. |  |  |
| 3. |  |  |

**(B5) Over the past month, have you been prescribed any medications through the NHS?**

1. **Yes**
2. **No**

**If ‘Yes’, please list any medications you were prescribed through the NHS over the past month:**

| **Medication (name/type)** | **Usual dose** | **Number of times daily** | **Length of course (Number of days)** | **Covid-related? (Yes/No)** |
| --- | --- | --- | --- | --- |
| *EXAMPLE: Paracetamol* | *2 tablets* | *1* | *10* | *Yes* |
|  |  |  |  |  |
|  |  |  |  |  |
|  |  |  |  |  |
|  |  |  |  |  |
|  |  |  |  |  |

**(B6) Over the past month, have you or your family incurred any expenses as a result of your Covid symptoms, other than those incurred for attending Long Covid clinic services?**

1. Yes
2. No

**If ‘Yes’, please list the expenditures below:**

| **Expense type** | **Please provide detail:** | **Cost to you or your family over the past month** |
| --- | --- | --- |
| *EXAMPLE: Travel costs* | *Bus to 1 appointment with cardiologist and 4 physiotherapy sessions* | *£15* |
| Private consultant fees |  | £ |
| Travel costs |  | £ |
| Childcare costs |  | £ |
| Home modifications |  | £ |
| Equipment (e.g., mobility aid) |  | £ |
| Alternative treatments |  | £ |
| Prescription medicines |  | £ |
| Medicines purchased over the counter |  | £ |
| Other private expenses, please specify below: | | |
| 1. |  | £ |
| 2. |  | £ |
| 3. |  | £ |

**(B7) Over the past month, how would you describe the overall impact of these private expenses on your personal finances?**

1. Minimal impact
2. Small impact
3. Moderate impact
4. Large impact
5. Catastrophic impact

**Caregiver role**

**(C1) Over the past month, have you received any help from unpaid caregivers for Covid symptoms?**

1. Yes
2. No

**If ‘Yes’, please describe the caregivers and type of unpaid help you received:**

|  | **Relationship** (e.g., partner, child, sibling) | **Help type** (e.g., housework, attending Covid services) | **Hours of help per week** |
| --- | --- | --- | --- |
| *EXAMPLE: Caregiver 1* | *Partner* | *Shopping, Attending Long Covid clinics* | *10* |
| Caregiver 1 |  |  |  |
| Caregiver 2 |  |  |  |
| Caregiver 3 |  |  |  |

**(C2) Over the past month, did your caregiver(s) reduce hours of paid/unpaid work to support you for your Covid symptoms?**

1. Yes
2. No

**If ‘Yes’, please tells us how many work hours per week were reduced:**

|  | **Work hours reduced per week:** | |
| --- | --- | --- |
|  | **Paid work:** | **Unpaid work:** |
| *EXAMPLE: Caregiver 1* | *10* |  |
| Caregiver 1 |  |  |
| Caregiver 2 |  |  |
| Caregiver 3 |  |  |

**Work status**

**(D0)** **Please describe your work status before contracting Covid:**

| **Work aspect** |  |
| --- | --- |
| *EXAMPLE:*  *Paid work* (including self-employed) | **Number of hours per week (state 0 if none):**  *40* |
| Paid work (including self-employed) | **Number of hours per week (state 0 if none):** |
| Unpaid work (e.g., childcare, education) | **Number of hours per week (state 0 if none):** |
| Performance at work | **Rate how any health problems affected your work performance before Covid (0 = very little, 100 = profoundly, leave blank if you didn’t work):** |
| Work income | **Monthly income before Covid (state £0 if none):**  £_____________ per month |

**(D1)** **Please describe your current work status:**

| **Work aspect** |  |
| --- | --- |
| Paid work (including self-employed) | **Number of hours per week (state 0 if none):** |
| Unpaid work (e.g., childcare, education) | **Number of hours per week (state 0 if none):** |
| Performance at work | **Rate how Covid symptoms affected your work performance over the past month (0 = very little, 100 = profoundly, leave blank if you didn’t work):** |
| Work income | **Income over the past month (state £0 if none):**  £_____________ over the past month |
| Other aspects | **Describe any other ways in which Covid symptoms have affected your work status (e.g., job change, retirement):** |

# Sample representativeness

| **Table A1** Statistical tests for difference in characteristics between respondents and non-respondents. | | | |
| --- | --- | --- | --- |
| **Characteristic** | **Respondents (n=366)** | **Non-respondents (n=348)** | **Statistical test for two-sample difference** |
| Male N (%) | 118 (32.2) | 120 (34.7) | Test of proportions: *P*=0.487 |
| Mean age (SD) | 48.3 (11.7) | 48.0 (12.6) | *t*-test: *P*=0.756 |
| White ethnicity N (%^a^) | 288 (88.1) | 254 (85.8) | Test of proportions: *P*=0.402 |
| IMD quintile N (%^b^)  *Most deprived*  *2^nd^*  *3^rd^*  *4^th^*  *Least deprived* | 45 (20.5)  39 (17.7)  35 (15.9)  50 (22.7)  51 (23.2) | 19 (19.0)  6 (6.0)  20 (20.0)  26 (26.0)  29 (29.0) | Mann-Whitney U test: *P*=0.101 |
| Hospitalised for COVID-19 N (%) | 40 (10.9) | 29 (8.6) | Test of proportions: *P*=0.301 |
| ^a^ Percentage of those who reported ethnicity data.  ^b^ Percentage of those who reported IMD quintile data.  **Abbreviation:** IMD: index of multiple deprivation; SD: standard deviation | | | |

# EQ-5D-5L dimension responses

| **Table A2** EQ-5D-5L dimension responses by duration of Long COVID. | | | | | |
| --- | --- | --- | --- | --- | --- |
| **Dimension/level** | **LC duration <1 year (N=163)** | **LC duration 1-2 years (N=101)** | **LC duration >2 years (N=61)** | **LC duration unknown (N=41)** | **Total (N=366)** |
| **Mobility**  *No problems*  *Slight problems*  *Moderate problems*  *Severe problems*  *Unable*  *Missing* | 38 (23.3)  46 (28.2)  45 (27.6)  18 (11.0)  0 (0.0)  16 (9.9) | 26 (25.7)  26 (25.7)  21 (20.8)  14 (13.9)  0 (0.0)  14 (13.9) | 10 (16.4)  17 (27.9)  12 (19.7)  15 (24.6)  1 (1.6)  6 (9.8) | 15 (36.6)  10 (24.4)  8 (19.5)  3 (7.3)  0 (0.0)  5 (12.2) | 89 (24.3)  99 (27.0)  86 (23.5)  50 (13.7)  1 (0.3)  41 (11.2) |
| **Self-care**  *No problems*  *Slight problems*  *Moderate problems*  *Severe problems*  *Unable*  *Missing* | 83 (50.9)  32 (19.6)  27 (16.6)  5 (3.1)  0 (0.0)  16 (9.8) | 51 (50.5)  17 (16.8)  15 (14.8)  4 (4.0)  0 (0.0)  14 (13.9) | 24 (39.4)  13 (21.3)  13 (21.3)  5 (8.2)  0 (0.0)  6 (9.8) | 22 (53.7)  8 (19.5)  6 (14.6)  0 (0.0)  0 (0.0)  5 (12.2) | 180 (49.2)  70 (19.1)  61 (16.7)  14 (3.8)  0 (0.0)  41 (11.2) |
| **Usual activities**  *No problems*  *Slight problems*  *Moderate problems*  *Severe problems*  *Unable*  *Missing* | 13 (8.0)  34 (20.8)  58 (35.6)  36 (22.1)  6 (3.7)  16 (9.8) | 12 (11.9)  20 (19.8)  31 (30.7)  18 (17.8)  6 (5.9)  14 (13.9) | 2 (3.3)  12 (19.7)  20 (32.8)  14 (22.9)  7 (11.5)  6 (9.8) | 6 (14.6)  14 (34.1)  9 (22.0)  4 (9.8)  3 (7.3)  5 (12.2) | 33 (9.0)  80 (21.9)  118 (32.2)  72 (19.7)  22 (6.0)  41 (11.2) |
| **Pain/discomfort**  *No pain*  *Slight pain*  *Moderate pain*  *Severe pain*  *Extreme pain*  *Missing* | 22 (13.5)  45 (27.6)  50 (30.7)  27 (16.6)  3 (1.8)  16 (9.8) | 15 (14.8)  23 (22.8)  32 (31.7)  15 (14.8)  2 (2.0)  14 (13.9) | 4 (6.7)  16 (26.2)  18 (29.5)  11 (18.0)  6 (9.8)  6 (9.8) | 5 (12.2)  16 (39.0)  10 (24.4)  5 (12.2)  0 (0.0)  5 (12.2) | 46 (12.6)  100 (27.3)  110 (30.1)  58 (15.8)  11 (3.0)  41 (11.2) |
| **Anxiety/depression**  *Not anxious*  *Slightly anxious*  *Moderately anxious*  *Severely anxious*  *Extremely anxious*  *Missing* | 31 (19.0)  57 (35.0)  37 (22.7)  17 (10.4)  5 (3.1)  16 (9.8) | 19 (18.8)  25 (24.8)  26 (25.7)  11 (10.9)  6 (5.9)  14 (13.9) | 8 (13.1)  16 (26.2)  19 (31.2)  8 (13.1)  4 (6.6)  6 (9.8) | 8 (19.5)  13 (31.7)  9 (21.9)  4 (9.8)  2 (4.9)  5 (12.2) | 66 (18.0)  111 (30.3)  91 (24.9)  40 (10.9)  17 (4.7)  41 (11.2) |
| **Abbreviation:** LC: Long COVID | | | | | |

# Productivity impact

**Figure A1** Change in paid work status/hours from the pre-infection period to the previous month for subgroup with Long COVID duration less than one year (N=163).

**Figure A2** Change in paid work status/hours from the pre-infection period to the previous month for subgroup with Long COVID duration between one and two years (N=101).

**Figure A3** Change in paid work status/hours from the pre-infection period to the previous month for subgroup with Long COVID duration longer than two years (N=61).

# Multivariate analyses

| **Table A3** Multivariate analysis of change in paid work hours. | | | | | | | | |
| --- | --- | --- | --- | --- | --- | --- | --- | --- |
| Model: multilevel linear regression^a,b^ | **(1) Demographics [N=270]** | | **(2) COVID-19 + (1) [N=266]** | | **(3) PROMs + (2) [N=209]** | | **(4) *P*<0.1 variables in (3) [N=250]** | |
|  | Co. (SE) | *P*-value | Co. (SE) | *P*-value | Co. (SE) | *P*-value | Co. (SE) | *P*-value |
| Constant | -5.926 (5.804) | 0.307 | -1.787 (6.346) | 0.778 | 2.010 (6.983) | 0.773 | -1.084 (4.547) | 0.812 |
| Pre-infection period paid work hours | **-0.528^¶^ (0.095)** | **<0.001** | **-0.552^¶^ (0.092)** | **<0.001** | **-0.679^¶^ (0.097)** | **<0.001** | **-0.659^¶^ (0.089)** | **<0.001** |
| LC duration (ref: <1 year) |  |  |  |  |  |  |  |  |
| *1-2 years* | 3.238 (2.233) | 0.147 | 1.508 (2.236) | 0.500 | 1.201 (2.422) | 0.620 | 2.674 (2.199) | 0.224 |
| *>2 years* | 2.814 (2.753) | 0.307 | 1.870 (2.712) | 0.490 | 4.490 (2.745) | 0.099 | **5.225* (2.576)** | **0.043** |
| *Missing* | 4.159 (3.467) | 0.230 | 3.302 (3.405) | 0.332 | 1.588 (3.683) | 0.666 | 3.202 (3.069) | 0.297 |
| Male (ref: female) | -1.809 (2.242) | 0.420 | -1.530 (2.209) | 0.488 | -3.152 (2.357) | 0.181 |  |  |
| Age group (ref: <35) |  |  |  |  |  |  |  |  |
| *35-44 years* | 1.625 (3.107) | 0.601 | 1.382 (3.037) | 0.649 | -0.751 (3.188) | 0.814 |  |  |
| *45-54 years* | 4.460 (2.978) | 0.134 | 3.299 (2.898) | 0.255 | 0.708 (2.997) | 0.813 |  |  |
| *55-64 years* | 3.151 (3.242) | 0.331 | 2.536 (3.135) | 0.419 | -1.436 (3.213) | 0.655 |  |  |
| *≥65 years* | -3.733 (6.161) | 0.545 | -6.039 (5.934) | 0.309 | -9.978 (6.490) | 0.124 |  |  |
| White ethnicity (ref: minority ethnic) | -2.889 (2.989) | 0.334 | -0.497 (2.936) | 0.865 | 0.229 (3.252) | 0.944 |  |  |
| IMD quintile (ref.: most deprived) |  |  |  |  |  |  |  |  |
| *2^nd^* | 3.325 (3.999) | 0.406 | 3.823 (3.902) | 0.327 | 0.340 (4.219) | 0.936 |  |  |
| *3^rd^* | 7.401 (3.963) | 0.062 | 6.513 (3.935) | 0.098 | 3.674 (4.548) | 0.419 |  |  |
| *4^th^* | **12.550** (3.895)** | **0.001** | **12.885****  **(3.849)** | **0.001** | 6.640 (4.119) | 0.107 |  |  |
| *Least deprived* | 3.204 (3.538) | 0.365 | 2.238 (3.484) | 0.521 | 0.200 (3.906) | 0.959 |  |  |
| *Missing* | 6.157 (3.144) | 0.050 | **6.776* (3.419)** | **0.047** | 3.814 (3.878) | 0.325 |  |  |
| Industry group (ref: health and education) |  |  |  |  |  |  |  |  |
| *Financial, ICT & professional* | **8.440** (2.811)** | **0.003** | **7.846** (2.756)** | **0.004** | **6.251* (2.788)** | **0.025** | **5.001* (2.546)** | **0.049** |
| *Other industries* | 2.005 (2.625) | 0.445 | 1.843 (2.561) | 0.472 | 2.177 (2.534) | 0.390 | 0.068 (2.386) | 0.977 |
| *Missing* | -0.453 (2.527) | 0.858 | -0.506 (2.491) | 0.839 | 0.683 (2.837) | 0.810 | -0.427 (2.496) | 0.864 |
| Hospitalised for COVID-19 (ref: not hospitalised) |  |  | 1.293 (3.194) | 0.686 | 1.711 (3.740) | 0.647 |  |  |
| COVID-19 vaccination (ref: single-vaccinated) |  |  |  |  |  |  |  |  |
| *Double-vaccinated* |  |  | -1.912 (2.735) | 0.484 | -2.476 (2.859) | 0.386 |  |  |
| *Missing* |  |  | -1.402 (3.266) | 0.668 | -2.926 (3.487) | 0.401 |  |  |
| Secondary care use in prev. month (ref: no use) |  |  | -1.939 (2.226) | 0.384 | -1.633 (2.407) | 0.497 |  |  |
| Community health/social care use in prev. month (ref: no use) |  |  | **-8.262^¶^ (1.917)** | **<0.001** | **-7.277^¶^**  **(2.064)** | **<0.001** | **-6.948^¶^**  **(1.948)** | **<0.001** |
| Number of services received at LC clinic |  |  | 0.599 (0.852) | 0.482 | 0.834 (0.883) | 0.345 |  |  |
| EQ-5D-3L utility rescaled from 0-1 to 0-100 |  |  |  |  | **0.132** (0.051)** | **0.009** | **0.163^¶^ (0.042)** | **<0.001** |
| **Highest quartile for:** |  |  |  |  |  |  |  |  |
| Increase in C19-YRS(m) symptom severity |  |  |  |  | -1.165 (2.579) | 0.652 |  |  |
| Increase in C19-YRS(m) functional disability |  |  |  |  | -4.895 (2.830) | 0.084 | -4.694 (2.513) | 0.062 |
| Improvement in C19-YRS(m) overall health |  |  |  |  | **5.556* (2.470)** | **0.024** | **7.179** (2.227)** | **0.001** |
| C19-YRS(m) other symptoms |  |  |  |  | -1.166 (2.589) | 0.652 |  |  |
| ^a^ Participants are grouped by region: Birmingham; Cardiff; Hertfordshire; Leeds; Leicester; London; Newcastle; Oxford; Salford. Complete cases are used. Coefficients, standard errors, and *P*-values are reported to three decimal places.  ^b^ Statistical significance: * *P*<0.05; ** *P*<0.01; ^¶^ *P*<0.001  **Abbreviation:** Co.: coefficient; ICT: information and communication technology; IMD: index of multiple deprivation; LC: Long COVID; prev.: previous; PROM: patient-reported outcome measure; ref: reference; SE: standard error | | | | | | | | |

| **Table A4** Multivariate analysis of change in paid/unpaid work performance (higher score implying worse performance), having returned to paid/unpaid work. | | | | | | | | |
| --- | --- | --- | --- | --- | --- | --- | --- | --- |
| Model: multilevel linear regression^a,b^ | **(1) Demographics [N=141]** | | **(2) COVID-19 + (1) [N=139]** | | **(3) PROMs + (2) [N=115]** | | **(4) *P*<0.1 variables in (3) [N=131]** | |
|  | Co. (SE) | *P*-value | Co. (SE) | *P*-value | Co. (SE) | *P*-value | Co. (SE) | *P*-value |
| Constant | **46.37^¶^ (10.08)** | **<0.001** | **27.47* (11.53)** | **0.017** | **60.40^¶^ (13.47)** | **<0.001** | **83.78^¶^ (8.13)** | **<0.001** |
| Pre-infection period work performance (0-100) | **-0.84^¶^ (0.07)** | **<0.001** | **-0.84^¶^ (0.07)** | **<0.001** | **-0.97^¶^ (0.07)** | **<0.001** | **-0.91^¶^ (0.06)** | **<0.001** |
| Same or increased paid/unpaid work works (ref: reduced hours) | **-19.09^¶^ (4.24)** | **<0.001** | **-16.40^¶^ (4.01)** | **<0.001** | **-12.37** (4.33)** | **0.004** | **-12.97** (3.92)** | **0.001** |
| LC duration (ref: <1 year) |  |  |  |  |  |  |  |  |
| *1-2 years* | -2.33 (5.20) | 0.654 | 4.35 (5.07) | 0.391 | 0.95 (5.17) | 0.854 |  |  |
| *>2 years* | -2.65 (6.04) | 0.660 | -4.77 (5.74) | 0.406 | -6.93 (5.36) | 0.196 |  |  |
| *Missing* | -10.46 (7.71) | 0.175 | -8.13 (7.63) | 0.287 | -6.44 (8.53) | 0.450 |  |  |
| Male (ref: female) | 5.91 (4.85) | 0.222 | 8.09 (4.80) | 0.092 | -0.57 (5.26) | 0.914 |  |  |
| Age group (ref: <35) |  |  |  |  |  |  |  |  |
| *35-44 years* | 9.05 (6.55) | 0.167 | 6.98 (6.24) | 0.263 | 9.88 (6.09) | 0.105 |  |  |
| *45-54 years* | -8.41 (6.19) | 0.174 | -9.87 (5.83) | 0.090 | -8.62 (5.76) | 0.134 |  |  |
| *55-64 years* | -0.90 (7.27) | 0.902 | -3.43 (6.87) | 0.618 | 8.55 (6.81) | 0.209 |  |  |
| White ethnicity (ref: minority ethnic) | 11.85 (6.70) | 0.077 | 9.71 (6.75) | 0.150 | 10.74 (7.15) | 0.133 |  |  |
| IMD quintile (ref.: most deprived) |  |  |  |  |  |  |  |  |
| *2^nd^* | 12.68 (9.05) | 0.161 | 12.04 (8.55) | 0.159 | 8.79 (8.85) | 0.320 |  |  |
| *3^rd^* | 8.94 (8.56) | 0.296 | 13.13 (8.43) | 0.119 | 2.53 (9.24) | 0.785 |  |  |
| *4^th^* | **15.40* (7.81)** | **0.049** | **15.50* (7.62)** | **0.042** | 11.69 (8.16) | 0.152 |  |  |
| *Least deprived* | 4.68 (7.91) | 0.554 | 5.38 (7.67) | 0.484 | 7.68 (8.18) | 0.348 |  |  |
| *Missing* | 8.74 (6.99) | 0.211 | **14.55* (7.12)** | **0.041** | 7.21 (8.15) | 0.376 |  |  |
| Industry group (ref: health and education) |  |  |  |  |  |  |  |  |
| *Financial, ICT & professional* | 1.69 (5.35) | 0.752 | 1.39 (5.20) | 0.789 | 1.20 (4.90) | 0.807 |  |  |
| *Other industries* | -4.18 (5.78) | 0.469 | -4.53 (5.46) | 0.407 | -5.91 (5.46) | 0.279 |  |  |
| *Missing* | 2.68 (5.86) | 0.647 | 2.40 (5.59) | 0.667 | -4.89 (6.35) | 0.440 |  |  |
| Hospitalised for COVID-19 (ref: not hospitalised) |  |  | 1.97 (7.21) | 0.784 | 0.54 (8.42) | 0.949 |  |  |
| COVID-19 vaccination (ref: single-vaccinated) |  |  |  |  |  |  |  |  |
| *Double-vaccinated* |  |  | 12.51 (6.53) | 0.050 | 11.00 (6.25) | 0.078 | 7.56 (5.76) | 0.189 |
| *Missing* |  |  | 1.08 (7.43) | 0.884 | 8.35 (7.45) | 0.262 | 4.78 (6.03) | 0.428 |
| Secondary care use in prev. month (ref: no use) |  |  | **12.75** (4.67)** | **0.006** | **11.21* (4.80)** | 0.497 | **13.98** (4.65)** | **0.003** |
| Community health/social care use in prev. month (ref: no use) |  |  | **11.03** (4.20)** | **0.009** | 7.40 (4.17) | 0.076 | 2.95  (4.00) | 0.461 |
| Number of services received at LC clinic |  |  | 2.41 (1.89) | 0.201 | 2.91 (2.07) | 0.159 |  |  |
| EQ-5D-3L utility rescaled from 0-1 to 0-100 |  |  |  |  | **-0.44** (0.13)** | **0.001** | **-0.46^¶^ (0.10)** | **<0.001** |
| **Highest quartile for:** |  |  |  |  |  |  |  |  |
| Increase in C19-YRS(m) symptom severity |  |  |  |  | 0.69 (5.59) | 0.902 |  |  |
| Increase in C19-YRS(m) functional disability |  |  |  |  | 2.49 (6.28) | 0.692 |  |  |
| Improvement in C19-YRS(m) overall health |  |  |  |  | **-16.51** (5.04)** | **0.001** | **-12.10** (4.62)** | **0.009** |
| C19-YRS(m) other symptoms |  |  |  |  | 5.69 (5.83) | 0.329 |  |  |
| ^a^ Participants are grouped by region: Birmingham; Cardiff; Hertfordshire; Leeds; Leicester; London; Newcastle; Oxford; Salford. Complete cases are used. Coefficients and standard errors are reported to two decimal places and *P*-values to three decimal places.  ^b^ Statistical significance: * *P*<0.05; ** *P*<0.01; ^¶^ *P*<0.001  **Abbreviation:** Co.: coefficient; ICT: information and communication technology; IMD: index of multiple deprivation; LC: Long COVID; prev.: previous; PROM: patient-reported outcome measure; ref: reference; SE: standard error | | | | | | | | |

| **Table A5** Multivariate analysis of change in monthly work income. | | | | | | | | |
| --- | --- | --- | --- | --- | --- | --- | --- | --- |
| Model: multilevel linear regression^a,b^ | **(1) Demographics [N=169]** | | **(2) COVID-19 + (1) [N=165]** | | **(3) PROMs + (2) [N=130]** | | **(4) *P*<0.1 variables in (3) [N=154]** | |
|  | Co. (SE) | *P*-value | Co. (SE) | *P*-value | Co. (SE) | *P*-value | Co. (SE) | *P*-value |
| Constant | -56.1 (331.7) | 0.866 | 227.8 (427.4) | 0.594 | -24.2 (553.3) | 0.965 | -329.7 (241.1) | 0.171 |
| Pre-infection period monthly work income | -0.1 (0.06) | 0.195 | -0.1 (0.06) | 0.284 | **-0.2* (0.06)** | **0.010** | **-0.2** (0.06)** | **0.003** |
| LC duration (ref: <1 year) |  |  |  |  |  |  |  |  |
| *1-2 years* | -38.6 (153.2) | 0.801 | -101.5 (159.3) | 0.524 | -239.8 (187.7) | 0.202 | -32.4 (162.9) | 0.842 |
| *>2 years* | **-480.8** (183.4)** | **0.009** | **-542.0** (189.6)** | **0.004** | -396.1 (207.6) | 0.056 | -332.5 (189.2) | 0.079 |
| *Missing* | -269.5 (280.8) | 0.337 | -322.3 (283.2) | 0.255 | -141.8 (308.4) | 0.646 | 65.7 (233.2) | 0.778 |
| Male (ref: female) | **-391.4* (164.3)** | **0.017** | **-428.4* (171.2)** | **0.012** | **-555.1** (201.2)** | **0.006** | -249.1 (158.1) | 0.115 |
| Age group (ref: <35) |  |  |  |  |  |  |  |  |
| *35-44 years* | 40.8 (212.1) | 0.847 | 17.5 (215.7) | 0.935 | 197.9 (234.5) | 0.399 |  |  |
| *45-54 years* | 97.0 (205.4) | 0.637 | 132.8 (205.0) | 0.517 | 171.1 (217.5) | 0.432 |  |  |
| *55-64 years* | 37.0 (229.0) | 0.872 | 84.7 (228.4) | 0.711 | 116.2 (243.1) | 0.633 |  |  |
| *≥65 years* | 233.1 (396.6) | 0.557 | 262.2 (401.1) | 0.513 | 24.7 (419.1) | 0.953 |  |  |
| White ethnicity (ref: minority ethnic) | -198.0 (248.2) | 0.425 | -210.9 (259.4) | 0.416 | -537.9 (345.9) | 0.120 |  |  |
| IMD quintile (ref.: most deprived) |  |  |  |  |  |  |  |  |
| *2^nd^* | -209.5 (254.8) | 0.411 | -199.4 (256.8) | 0.437 | -18.1 (284.7) | 0.949 |  |  |
| *3^rd^* | -26.0 (263.3) | 0.922 | -124.8 (281.2) | 0.657 | -362.0 (338.1) | 0.284 |  |  |
| *4^th^* | 330.5 (269.7) | 0.220 | 219.2 (280.7) | 0.435 | 211.5 (320.3) | 0.509 |  |  |
| *Least deprived* | -107.1 (241.9) | 0.658 | -124.8 (246.5) | 0.613 | 143.4 (290.7) | 0.622 |  |  |
| *Missing* | 235.9 (217.0) | 0.277 | 352.0 (264.1) | 0.183 | 470.0 (332.5) | 0.157 |  |  |
| Industry group (ref: health and education) |  |  |  |  |  |  |  |  |
| *Financial, ICT & professional* | **388.4* (180.7)** | **0.032** | **389.0* (181.5)** | **0.032** | **472.4* (190.1)** | **0.013** | **350.5* (176.1)** | **0.046** |
| *Other industries* | 92.3 (184.5) | 0.617 | 79.4 (186.4) | 0.670 | 57.2 (198.2) | 0.773 | -75.3 (179.6) | 0.675 |
| *Missing* | -248.4 (179.8) | 0.167 | -351.9 (187.0) | 0.060 | -329.0 (235.5) | 0.162 | -243.4 (194.4) | 0.211 |
| Hospitalised for COVID-19 (ref: not hospitalised) |  |  | 61.8 (210.1) | 0.769 | 515.9 (210.1) | 0.058 | 34.7 (231.7) | 0.881 |
| COVID-19 vaccination (ref: single-vaccinated) |  |  |  |  |  |  |  |  |
| *Double-vaccinated* |  |  | -298.8 (210.2) | 0.155 | -208.7 (250.5) | 0.405 |  |  |
| *Missing* |  |  | -335.2 (264.2) | 0.205 | -356.7 (321.1) | 0.267 |  |  |
| Secondary care use in prev. month (ref: no use) |  |  | **-369.0* (159.2)** | **0.020** | **-466.6** (169.3)** | **0.006** | -296.4 (153.8) | 0.054 |
| Community health/social care use in prev. month (ref: no use) |  |  | 29.8 (138.5) | 0.830 | 60.6 (152.0) | 0.690 |  |  |
| Number of services received at LC clinic |  |  | 53.5 (72.4) | 0.460 | 152.1 (81.0) | 0.061 | 71.8 (64.3) | 0.264 |
| EQ-5D-3L utility rescaled from 0-1 to 0-100 |  |  |  |  | **8.3***  **(3.9)** | **0.031** | **8.0****  **(2.8)** | **0.004** |
| **Highest quartile for:** |  |  |  |  |  |  |  |  |
| Increase in C19-YRS(m) symptom severity |  |  |  |  | **-399.9* (192.7)** | **0.038** | -273.8 (168.2) | 0.104 |
| Increase in C19-YRS(m) functional disability |  |  |  |  | 228.7 (208.6) | 0.273 |  |  |
| Improvement in C19-YRS(m) overall health |  |  |  |  | 122.7 (194.9) | 0.529 |  |  |
| C19-YRS(m) other symptoms |  |  |  |  | 93.6 (208.5) | 0.653 |  |  |
| ^a^ Participants are grouped by region: Birmingham; Cardiff; Hertfordshire; Leeds; Leicester; London; Newcastle; Oxford; Salford. Complete cases are used. Coefficients and standard errors are reported to one decimal place and *P*-values to three decimal places.  ^b^ Statistical significance: * *P*<0.05; ** *P*<0.01; ^¶^ *P*<0.001  **Abbreviation:** Co.: coefficient; ICT: information and communication technology; IMD: index of multiple deprivation; LC: Long COVID; prev.: previous; PROM: patient-reported outcome measure; ref: reference; SE: standard error | | | | | | | | |

# EQ-5D-5L dimension responses as covariates

| **Table A6** Multivariate analysis of returning to same/higher paid work hours as pre-COVID-19, with EQ-5D-5L dimensions as covariates. | | | | | | | | | | |
| --- | --- | --- | --- | --- | --- | --- | --- | --- | --- | --- |
| **Model: multilevel logistic regression^a,b^** | **Mobility [N=249]** | | **Self-care [N=250]** | | **Usual activities [N=250]** | | **Pain/discomfort [N=243]** | | **Anxiety/depression [N=250]** | |
|  | **OR (SE)** | ***P*-value** | **OR (SE)** | ***P*-value** | **OR (SE)** | ***P*-value** | **OR (SE)** | ***P*-value** | **OR (SE)** | ***P*-value** |
| Constant | 1.557 (0.574) | 0.229 | 0.883 (0.242) | 0.649 | 1.307 (0.620) | 0.572 | 0.681 (0.294) | 0.374 | 0.960 (0.372) | 0.916 |
| LC duration (ref: <1 year) |  |  |  |  |  |  |  |  |  |  |
| *1-2 years* | **2.487* (0.914)** | **0.013** | **2.737** (0.991)** | **0.005** | **2.731** (1.003)** | **0.006** | **2.779** (1.019)** | **0.005** | **2.571** (0.917)** | **0.008** |
| *>2 years* | 2.173 (0.999) | 0.092 | 2.384 (1.075) | 0.054 | **2.446* (1.116)** | **0.050** | **2.549* (1.199)** | **0.047** | 2.305 (1.024) | 0.060 |
| *Missing* | 1.654 (0.836) | 0.319 | 2.013 (0.999) | 0.159 | 1.638 (0.825) | 0.327 | 1.930 (0.947) | 0.180 | 2.172 (1.075) | 0.117 |
| Community health/social care use in prev. month (ref: no use) | **0.417* (0.142)** | **0.010** | **0.469* (0.158)** | **0.025** | **0.487* (0.164)** | **0.033** | **0.471* (0.160)** | **0.027** | **0.405** (0.133)** | **0.006** |
| Highest quartile for Increase in C19-YRS(m) functional disability | **0.183** (0.110)** | **0.005** | **0.223** (0.122)** | **0.006** | **0.221** (0.127)** | **0.008** | **0.183** (0.099)** | **0.002** | **0.129^¶^ (0.066)** | **<0.001** |
| **EQ-5D-5L dimensions** |  |  |  |  |  |  |  |  |  |  |
| Mobility (ref: No problems) |  |  |  |  |  |  |  |  |  |  |
| *Slight problems* | **0.427* (0.159)** | **0.022** |  |  |  |  |  |  |  |  |
| *Moderate problems* | **0.201^¶^ (0.088)** | **<0.001** |  |  |  |  |  |  |  |  |
| *Severe problems* | 0.328 (0.225) | 0.104 |  |  |  |  |  |  |  |  |
| *Unable* | Empty |  |  |  |  |  |  |  |  |  |
| Self-care (ref: No problems) |  |  |  |  |  |  |  |  |  |  |
| *Slight problems* |  |  | **0.406* (0.158)** | **0.020** |  |  |  |  |  |  |
| *Moderate problems* |  |  | **0.181* (0.123)** | **0.012** |  |  |  |  |  |  |
| *Severe problems* |  |  | 0.243 (0.279) | 0.219 |  |  |  |  |  |  |
| *Unable* |  |  | Empty |  |  |  |  |  |  |  |
| Usual activities (ref: No problems) |  |  |  |  |  |  |  |  |  |  |
| *Slight problems* |  |  |  |  | 0.967 (0.497) | 0.948 |  |  |  |  |
| *Moderate problems* |  |  |  |  | **0.361* (0.180)** | **0.042** |  |  |  |  |
| *Severe problems* |  |  |  |  | **0.178** (0.110)** | **0.005** |  |  |  |  |
| *Unable* |  |  |  |  | 0.213 (0.213) | 0.122 |  |  |  |  |
| Pain/discomfort (ref: No pain) |  |  |  |  |  |  |  |  |  |  |
| *Slight pain* |  |  |  |  |  |  | 1.603 (0.705) | 0.283 |  |  |
| *Moderate pain* |  |  |  |  |  |  | 0.678 (0.310) | 0.396 |  |  |
| *Severe pain* |  |  |  |  |  |  | 0.454 (0.307) | 0.242 |  |  |
| *Extreme pain* |  |  |  |  |  |  | Empty |  |  |  |
| Anxiety/depression (ref: Not anxious) |  |  |  |  |  |  |  |  |  |  |
| *Slightly anxious* |  |  |  |  |  |  |  |  | 0.864 (0.336) | 0.706 |
| *Moderately anxious* |  |  |  |  |  |  |  |  | 0.563 (0.240) | 0.178 |
| *Severely anxious* |  |  |  |  |  |  |  |  | 0.602 (0.344) | 0.375 |
| *Extremely anxious* |  |  |  |  |  |  |  |  | 0.167 (0.192) | 0.119 |
| ^a^ See model (4) in Table 5 for selection of explanatory variables. Participants are grouped by region: Birmingham; Cardiff; Hertfordshire; Leeds; Leicester; London; Newcastle; Oxford; Salford. Complete cases are used. Odds ratios, standard errors, and *P*-values are reported to three decimal places.  ^b^ Statistical significance: * *P*<0.05; ** *P*<0.01; ^¶^ *P*<0.001.  **Abbreviation:** LC: Long COVID; OR: odds ratio; ref: reference; SE: standard error. | | | | | | | | | | |

| **Table A7** Multivariate analysis of change in paid work hours, with EQ-5D-5L dimensions as covariates. | | | | | | | | | | |
| --- | --- | --- | --- | --- | --- | --- | --- | --- | --- | --- |
| **Model: multilevel linear regression^a,b^** | **Mobility [N=236]** | | **Self-care [N=236]** | | **Usual activities [N=236]** | | **Pain/discomfort [N=236]** | | **Anxiety/depression [N=236]** | |
|  | **Co. (SE)** | ***P*-value** | **Co. (SE)** | ***P*-value** | **Co. (SE)** | ***P*-value** | **Co. (SE)** | ***P*-value** | **Co. (SE)** | ***P*-value** |
| Constant | **12.225** (3.988)** | **0.002** | **12.260** (3.706)** | **0.001** | **12.061** (4.392)** | **0.006** | **11.866** (4.471)** | **0.008** | **9.453* (3.989)** | **0.018** |
| Pre-infection period paid work hours | **-0.643^¶^ (0.089)** | **<0.001** | **-0.686^¶^ (0.092)** | **<0.001** | **-0.629^¶^ (0.088)** | **<0.001** | **-0.660^¶^ (0.091)** | **<0.001** | **-0.673^¶^ (0.092)** | **<0.001** |
| LC duration (ref: <1 year) |  |  |  |  |  |  |  |  |  |  |
| *1-2 years* | 2.209 (2.205) | 0.316 | 2.465 (2.189) | 0.260 | 2.382 (2.166) | 0.271 | 2.508 (2.230) | 0.261 | 2.645 (2.244) | 0.238 |
| *>2 years* | 4.154 (2.579) | 0.107 | 4.676 (2.588) | 0.071 | 4.821 (2.525) | 0.056 | 4.452 (2.623) | 0.090 | 4.125 (2.601) | 0.113 |
| *Missing* | 2.510 (3.115) | 0.420 | 4.355 (3.057) | 0.154 | 3.280 (3.050) | 0.282 | 2.798 (3.127) | 0.371 | 4.442 (3.122) | 0.155 |
| Industry group (ref: health and education) |  |  |  |  |  |  |  |  |  |  |
| *Financial, ICT & professional* | 4.877 (2.562) | 0.057 | 3.811 (2.548) | 0.135 | **5.360* (2.515)** | **0.033** | 4.554 (2.613) | 0.081 | **5.728* (2.604)** | **0.028** |
| *Other industries* | -0.393 (2.391) | 0.869 | -0.940 (2.364) | 0.691 | 0.108 (2.365) | 0.963 | -0.181 (2.420) | 0.940 | -0.633 (2.418) | 0.793 |
| *Missing* | -0.408 (2.522) | 0.871 | -1.441 (2.474) | 0.560 | -1.242 (2.465) | 0.614 | -0.287 (2.562) | 0.911 | -0.071 (2.572) | 0.978 |
| Community health/social care use in prev. month (ref: no use) | **-7.587^¶^ (1.935)** | **<0.001** | **-7.167^¶^ (1.928)** | **<0.001** | **-6.703^¶^ (1.914)** | **<0.001** | **-7.514^¶^ (1.967)** | **<0.001** | **-8.128^¶^ (1.948)** | **<0.001** |
| Highest quartile for increase in C19-YRS(m) functional disability | **-5.430* (2.672)** | **0.042** | **-4.957* (2.460)** | **0.044** | -3.136 (2.630) | 0.233 | **-6.173* (2.443)** | **0.012** | **-8.673^¶^ (2.236)** | **<0.001** |
| Highest quartile for improvement in C19-YRS(m) overall health | **7.346** (2.272)** | **0.001** | **6.996** (2.218)** | **0.002** | **6.684** (2.214)** | **0.003** | **7.766** (2.261)** | **0.001** | **6.795** (2.276)** | **0.003** |
| **EQ-5D-5L dimensions** |  |  |  |  |  |  |  |  |  |  |
| Mobility (ref: No problems) |  |  |  |  |  |  |  |  |  |  |
| *Slight problems* | -3.467 (2.368) | 0.143 |  |  |  |  |  |  |  |  |
| *Moderate problems* | **-6.529* (2.643)** | **0.013** |  |  |  |  |  |  |  |  |
| *Severe problems* | **-10.410** (3.621)** | **0.004** |  |  |  |  |  |  |  |  |
| *Unable* | **-30.002* (14.133)** | **0.034** |  |  |  |  |  |  |  |  |
| Self-care (ref: No problems) |  |  |  |  |  |  |  |  |  |  |
| *Slight problems* |  |  | -3.067 (2.342) | 0.190 |  |  |  |  |  |  |
| *Moderate problems* |  |  | **-11.943^¶^ (2.928)** | **<0.001** |  |  |  |  |  |  |
| *Severe problems* |  |  | -11.082 (5.666) | 0.050 |  |  |  |  |  |  |
| *Unable* |  |  | Empty |  |  |  |  |  |  |  |
| Usual activities (ref: No problems) |  |  |  |  |  |  |  |  |  |  |
| *Slight problems* |  |  |  |  | -0.202 (3.237) | 0.950 |  |  |  |  |
| *Moderate problems* |  |  |  |  | -5.343 (3.160) | 0.091 |  |  |  |  |
| *Severe problems* |  |  |  |  | **-12.616** (3.632)** | **0.001** |  |  |  |  |
| *Unable* |  |  |  |  | **-15.202****  **(4.895)** | **0.002** |  |  |  |  |
| Pain/discomfort (ref: No pain) |  |  |  |  |  |  |  |  |  |  |
| *Slight pain* |  |  |  |  |  |  | -0.410 (2.903) | 0.888 |  |  |
| *Moderate pain* |  |  |  |  |  |  | -5.302 (2.995) | 0.077 |  |  |
| *Severe pain* |  |  |  |  |  |  | **-9.344 (3.807)** | **0.014** |  |  |
| *Extreme pain* |  |  |  |  |  |  | -10.057 (6.143) | 0.102 |  |  |
| Anxiety/depression (ref: Not anxious) |  |  |  |  |  |  |  |  |  |  |
| *Slightly anxious* |  |  |  |  |  |  |  |  | 2.829 (2.496) | 0.257 |
| *Moderately anxious* |  |  |  |  |  |  |  |  | -1.599 (2.660) | 0.548 |
| *Severely anxious* |  |  |  |  |  |  |  |  | -0.898 (3.517) | 0.798 |
| *Extremely anxious* |  |  |  |  |  |  |  |  | -9.053 (4.742) | 0.056 |
| ^a^ See model (4) in Table A3 for selection of explanatory variables. Participants are grouped by region: Birmingham; Cardiff; Hertfordshire; Leeds; Leicester; London; Newcastle; Oxford; Salford. Complete cases are used. Coefficients, standard errors, and *P*-values are reported to three decimal places.  ^b^ Statistical significance: * *P*<0.05; ** *P*<0.01; ^¶^ *P*<0.001.  **Abbreviation:** Co.: coefficient; LC: Long COVID; ref: reference; SE: standard error. | | | | | | | | | | |

| **Table A8** Multivariate analysis of change in work performance, with EQ-5D-5L dimensions as covariates. | | | | | | | | | | |
| --- | --- | --- | --- | --- | --- | --- | --- | --- | --- | --- |
| **Model: multilevel linear regression^a,b^** | **Mobility [N=131]** | | **Self-care [N=131]** | | **Usual activities [N=131]** | | **Pain/discomfort [N=131]** | | **Anxiety/depression [N=131]** | |
|  | **Co. (SE)** | ***P*-value** | **Co. (SE)** | ***P*-value** | **Co. (SE)** | ***P*-value** | **Co. (SE)** | ***P*-value** | **Co. (SE)** | ***P*-value** |
| Constant | **42.12^¶^ (7.29)** | **<0.001** | **56.85^¶^ (6.70)** | **<0.001** | **38.01^¶^ (8.01)** | **<0.001** | **54.05^¶^ (7.59)** | **<0.001** | **56.92^¶^ (7.64)** | **<0.001** |
| Pre-infection period work performance (0-100) | **-0.84^¶^ (0.06)** | **<0.001** | **-0.85^¶^ (0.07)** | **<0.001** | **-0.84^¶^ (0.06)** | **<0.001** | **-0.88^¶^ (0.06)** | **<0.001** | **-0.85^¶^ (0.07)** | **<0.001** |
| Same or increased paid/unpaid work works (ref: reduced hours) | **-12.41** (3.99)** | **0.002** | **-14.94^¶^ (4.24)** | **<0.001** | **-11.76** (3.88)** | **0.002** | **-13.54** (3.97)** | **0.001** | **-16.44^¶^ (4.10)** | **<0.001** |
| COVID-19 vaccination (ref: single-vaccinated) |  |  |  |  |  |  |  |  |  |  |
| *Double-vaccinated* | 7.87  (5.79) | 0.174 | 3.69  (6.15) | 0.549 | 3.85  (5.48) | 0.482 | 3.24  (5.82) | 0.577 | 3.94  (6.16) | 0.522 |
| *Missing* | 4.87  (6.05) | 0.421 | 2.01  (6.50) | 0.757 | 2.43  (5.74) | 0.672 | -0.50  (6.21) | 0.936 | 3.22  (6.48) | 0.619 |
| Secondary care use in prev. month (ref: no use) | **13.64** (4.66)** | **0.003** | **15.23** (4.96)** | **0.002** | **12.49** (4.62)** | **0.007** | **14.27** (4.73)** | **0.003** | **14.25** (5.00)** | **0.004** |
| Community health/social care use in prev. month (ref: no use) | 4.73  (4.03) | 0.240 | 3.14  (4.36) | 0.471 | 2.10  (3.84) | 0.584 | 2.76  (4.07) | 0.498 | 3.95  (4.31) | 0.359 |
| Highest quartile for improvement in C19-YRS(m) overall health | **-10.26***  **(4.666)** | **0.028** | **-12.00***  **(4.97)** | **0.016** | **-10.31* (4.47)** | **0.021** | **-12.83** (4.72)** | **0.007** | **-11.46* (4.96)** | **0.021** |
| **EQ-5D-5L dimensions** |  |  |  |  |  |  |  |  |  |  |
| Mobility (ref: No problems) |  |  |  |  |  |  |  |  |  |  |
| *Slight problems* | **17.88^¶^ (4.38)** | **<0.001** |  |  |  |  |  |  |  |  |
| *Moderate problems* | **16.97^**^ (5.48)** | **0.002** |  |  |  |  |  |  |  |  |
| *Severe problems* | **20.83^**^ (7.21)** | **0.004** |  |  |  |  |  |  |  |  |
| *Unable* | Empty |  |  |  |  |  |  |  |  |  |
| Self-care (ref: No problems) |  |  |  |  |  |  |  |  |  |  |
| *Slight problems* |  |  | 6.66  (5.07) | 0.189 |  |  |  |  |  |  |
| *Moderate problems* |  |  | 10.22  (8.50) | 0.229 |  |  |  |  |  |  |
| *Severe problems* |  |  | 9.59  (23.36) | 0.681 |  |  |  |  |  |  |
| *Unable* |  |  | Empty |  |  |  |  |  |  |  |
| Usual activities (ref: No problems) |  |  |  |  |  |  |  |  |  |  |
| *Slight problems* |  |  |  |  | **14.66* (6.06)** | **0.016** |  |  |  |  |
| *Moderate problems* |  |  |  |  | **20.91** (6.17)** | **0.001** |  |  |  |  |
| *Severe problems* |  |  |  |  | **40.59^¶^ (7.04)** | **<0.001** |  |  |  |  |
| *Unable* |  |  |  |  | 16.71 (11.79) | 0.157 |  |  |  |  |
| Pain/discomfort (ref: No pain) |  |  |  |  |  |  |  |  |  |  |
| *Slight pain* |  |  |  |  |  |  | -0.39  (5.46) | 0.943 |  |  |
| *Moderate pain* |  |  |  |  |  |  | **14.62***  **(5.85)** | **0.012** |  |  |
| *Severe pain* |  |  |  |  |  |  | **18.58***  **(8.25)** | **0.024** |  |  |
| *Extreme pain* |  |  |  |  |  |  | 28.49  (21.93) | 0.194 |  |  |
| Anxiety/depression (ref: Not anxious) |  |  |  |  |  |  |  |  |  |  |
| *Slightly anxious* |  |  |  |  |  |  |  |  | 1.35  (5.16) | 0.794 |
| *Moderately anxious* |  |  |  |  |  |  |  |  | 1.45  (5.90) | 0.806 |
| *Severely anxious* |  |  |  |  |  |  |  |  | 8.74  (7.58) | 0.248 |
| *Extremely anxious* |  |  |  |  |  |  |  |  | 14.83  (16.67) | 0.374 |
| ^a^ See model (4) in Table A4 for selection of explanatory variables. Participants are grouped by region: Birmingham; Cardiff; Hertfordshire; Leeds; Leicester; London; Newcastle; Oxford; Salford. Complete cases are used. Coefficients and standard errors are reported to two decimal places and *P*-values to three decimal places.  ^b^ Statistical significance: * *P*<0.05; ** *P*<0.01; ^¶^ *P*<0.001.  **Abbreviation:** Co.: coefficient; ref: reference; SE: standard error. | | | | | | | | | | |

| **Table A9** Multivariate analysis of change in monthly work income, with EQ-5D-5L dimensions as covariates. | | | | | | | | | | |
| --- | --- | --- | --- | --- | --- | --- | --- | --- | --- | --- |
| **Model: multilevel linear regression^a,b^** | **Mobility [N=154]** | | **Self-care [N=154]** | | **Usual activities [N=154]** | | **Pain/discomfort [N=154]** | | **Anxiety/depression [N=154]** | |
|  | **Co. (SE)** | ***P*-value** | **Co. (SE)** | ***P*-value** | **Co. (SE)** | ***P*-value** | **Co. (SE)** | ***P*-value** | **Co. (SE)** | ***P*-value** |
| Constant | 265.8 (234.5) | 0.257 | 214.4 (216.4) | 0.322 | 340.6 (306.8) | 0.267 | 220.4 (285.2) | 0.440 | 233.4 (247.5) | 0.345 |
| Pre-infection period monthly work income | **-0.1** (0.06)** | **0.009** | **-0.2***  **(0.06)** | **0.011** | **-0.1***  **(0.06)** | **0.017** | **-0.2****  **(0.06)** | **0.006** | **-0.1***  **(0.06)** | **0.016** |
| LC duration (ref: <1 year) |  |  |  |  |  |  |  |  |  |  |
| *1-2 years* | -67.4  (167.1) | 0.687 | -44.7  (166.4) | 0.788 | -45.9  (165.8) | 0.782 | 4.3  (164.8) | 0.979 | 50.3  (165.6) | 0.761 |
| *>2 years* | **-411.4* (193.0)** | **0.033** | **-384.4* (194.4)** | **0.048** | -341.6 (188.0) | 0.069 | -301.8 (192.4) | 0.117 | **-381.2* (188.1)** | **0.043** |
| *Missing* | -34.3 (244.4) | 0.888 | 52.4  (237.5) | 0.825 | 52.1  (236.1) | 0.825 | 108.4 (232.9) | 0.642 | 153.8 (235.5) | 0.514 |
| Male (ref: female) | -218.1 (162.2) | 0.179 | -243.3 (163.9) | 0.138 | -201.0 (161.4) | 0.213 | -289.6 (157.3) | 0.065 | -308.3 (162.1) | 0.057 |
| Industry group (ref: health and education) |  |  |  |  |  |  |  |  |  |  |
| *Financial, ICT & professional* | 330.2 (178.1) | 0.064 | 317.1 (180.9) | 0.080 | **349.6* (177.2)** | **0.049** | 334.1 (181.0) | 0.065 | **417.4* (178.1)** | **0.019** |
| *Other industries* | -83.8 (183.3) | 0.648 | -70.6 (183.4) | 0.700 | -86.0 (185.9) | 0.644 | 1.0  (181.7) | 0.995 | -42.2 (180.0) | 0.815 |
| *Missing* | -246.0 (200.9) | 0.221 | -267.6 (197.5) | 0.176 | -285.9 (195.2) | 0.143 | -194.5 (196.2) | 0.322 | -223.3 (197.0) | 0.257 |
| Hospitalised for COVID-19 (ref: not hospitalised) | 9.9  (234.3) | 0.966 | 4.5  (236.3) | 0.985 | -1.8  (231.6) | 0.994 | -35.7 (232.6) | 0.878 | -121.5 (237.5) | 0.609 |
| Secondary care use in prev. month (ref: no use) | -251.0 (157.5) | 0.111 | **-307.4* (156.4)** | **0.049** | -235.8 (157.4) | 0.134 | -281.1 (153.4) | 0.067 | -274.4 (155.2) | 0.077 |
| Number of services received at LC clinic | 70.2  (67.6) | 0.299 | 44.2  (65.2) | 0.498 | 48.7  (63.5) | 0.443 | 60.4  (63.9) | 0.344 | 54.4  (64.0) | 0.395 |
| Highest quartile for increase in C19-YRS(m) symptom severity | -323.2  (170.5) | 0.058 | **-346.1***  **(173.5)** | **0.046** | -274.3  (171.3) | 0.109 | **-330.6***  **(162.0)** | **0.041** | **-400.8***  **(159.3)** | **0.012** |
| **EQ-5D-5L dimensions** |  |  |  |  |  |  |  |  |  |  |
| Mobility (ref: No problems) |  |  |  |  |  |  |  |  |  |  |
| *Slight problems* | -167.9 (179.3) | 0.349 |  |  |  |  |  |  |  |  |
| *Moderate problems* | -240.0 (197.4) | 0.224 |  |  |  |  |  |  |  |  |
| *Severe problems* | **-501.2* (216.8)** | **0.021** |  |  |  |  |  |  |  |  |
| *Unable* | -810.1 (854.5) | 0.343 |  |  |  |  |  |  |  |  |
| Self-care (ref: No problems) |  |  |  |  |  |  |  |  |  |  |
| *Slight problems* |  |  | -118.8 (181.0) | 0.512 |  |  |  |  |  |  |
| *Moderate problems* |  |  | -335.9 (199.5) | 0.092 |  |  |  |  |  |  |
| *Severe problems* |  |  | -94.2 (356.6) | 0.792 |  |  |  |  |  |  |
| *Unable* |  |  | Empty |  |  |  |  |  |  |  |
| Usual activities (ref: No problems) |  |  |  |  |  |  |  |  |  |  |
| *Slight problems* |  |  |  |  | -121.6 (274.1) | 0.657 |  |  |  |  |
| *Moderate problems* |  |  |  |  | -316.5 (273.0) | 0.246 |  |  |  |  |
| *Severe problems* |  |  |  |  | -488.5 (304.1) | 0.108 |  |  |  |  |
| *Unable* |  |  |  |  | **-749.3* (334.4)** | **0.025** |  |  |  |  |
| Pain/discomfort (ref: No pain) |  |  |  |  |  |  |  |  |  |  |
| *Slight pain* |  |  |  |  |  |  | -30.3 (212.1) | 0.886 |  |  |
| *Moderate pain* |  |  |  |  |  |  | -135.2 (217.1) | 0.534 |  |  |
| *Severe pain* |  |  |  |  |  |  | **-718.9* (281.3)** | **0.011** |  |  |
| *Extreme pain* |  |  |  |  |  |  | -790.1 (457.2) | 0.084 |  |  |
| Anxiety/depression (ref: Not anxious) |  |  |  |  |  |  |  |  |  |  |
| *Slightly anxious* |  |  |  |  |  |  |  |  | -171.1 (190.6) | 0.369 |
| *Moderately anxious* |  |  |  |  |  |  |  |  | -339.6 (202.2) | 0.093 |
| *Severely anxious* |  |  |  |  |  |  |  |  | 86.1  (262.7) | 0.743 |
| *Extremely anxious* |  |  |  |  |  |  |  |  | **-752.8* (320.5)** | **0.019** |
| ^a^ See model (4) in Table A5 for selection of explanatory variables. Participants are grouped by region: Birmingham; Cardiff; Hertfordshire; Leeds; Leicester; London; Newcastle; Oxford; Salford. Complete cases are used. Coefficients and standard errors are reported to one decimal place and *P*-values to three decimal places.  ^b^ Statistical significance: * *P*<0.05; ** *P*<0.01; ^¶^ *P*<0.001.  **Abbreviation:** Co.: coefficient; LC: Long COVID; ref: reference; SE: standard error. | | | | | | | | | | |
| **Table A10** Multivariate analysis of receiving any informal care, with EQ-5D-5L dimensions as covariates. | | | | | | | | | | |
| **Model: multilevel logistic regression^a,b^** | **Mobility [N=300]** | | **Self-care [N=301]** | | **Usual activities [N=301]** | | **Pain/discomfort [N=301]** | | **Anxiety/depression [N=301]** | |
|  | **OR (SE)** | ***P*-value** | **OR (SE)** | ***P*-value** | **OR (SE)** | ***P*-value** | **OR (SE)** | ***P*-value** | **OR (SE)** | ***P*-value** |
| Constant | **0.142^¶^ (0.053)** | **<0.001** | **0.186^¶^ (0.051)** | **<0.001** | **0.034^**^ (0.035)** | **0.001** | **0.159^¶^ (0.073)** | **<0.001** | **0.144^¶^ (0.059)** | **<0.001** |
| LC duration (ref: <1 year) |  |  |  |  |  |  |  |  |  |  |
| *1-2 years* | **0.451* (0.175)** | **0.040** | **0.460* (0.179)** | **0.046** | **0.452* (0.175)** | **0.041** | **0.431* (0.167)** | **0.030** | **0.398* (0.160)** | **0.022** |
| *>2 years* | 0.637 (0.257) | 0.264 | 0.579 (0.232) | 0.172 | 0.638 (0.252) | 0.256 | 0.614 (0.248) | 0.227 | 0.588 (0.238) | 0.190 |
| *Missing* | 0.607 (0.313) | 0.332 | 0.564 (0.286) | 0.259 | 0.703 (0.368) | 0.501 | 0.622 (0.316) | 0.349 | 0.466 (0.246) | 0.148 |
| Hospitalised for COVID-19 (ref: not hospitalised) | 2.101 (1.028) | 0.129 | 2.159 (1.064) | 0.118 | 2.590 (1.271) | 0.052 | 2.452 (1.165) | 0.059 | 2.332 (1.187) | 0.096 |
| Community health/social care use in prev. month (ref: no use) | **2.569** (0.742)** | **0.001** | **2.464** (0.712)** | **0.002** | **2.256** (0.667)** | **0.006** | **2.427** (0.712)** | **0.003** | **2.490** (0.728)** | **0.002** |
| Highest quartile for increase in C19-YRS(m) symptom severity | **3.091** (1.012)** | **0.001** | **3.167^¶^ (1.035)** | **<0.001** | **3.466^¶^ (1.164)** | **<0.001** | **3.349^¶^ (1.079)** | **<0.001** | **4.193^¶^ (1.387)** | **<0.001** |
| **EQ-5D-5L dimensions** |  |  |  |  |  |  |  |  |  |  |
| Mobility (ref: No problems) |  |  |  |  |  |  |  |  |  |  |
| *Slight problems* | 1.418 (0.598) | 0.407 |  |  |  |  |  |  |  |  |
| *Moderate problems* | **2.849* (1.169)** | **0.011** |  |  |  |  |  |  |  |  |
| *Severe problems* | **4.335** (2.061)** | **0.002** |  |  |  |  |  |  |  |  |
| *Unable* | Empty |  |  |  |  |  |  |  |  |  |
| Self-care (ref: No problems) |  |  |  |  |  |  |  |  |  |  |
| *Slight problems* |  |  | 1.781 (0.644) | 0.111 |  |  |  |  |  |  |
| *Moderate problems* |  |  | **2.993** (1.093)** | **0.003** |  |  |  |  |  |  |
| *Severe problems* |  |  | 4.001 (2.865) | 0.053 |  |  |  |  |  |  |
| *Unable* |  |  | Empty |  |  |  |  |  |  |  |
| Usual activities (ref: No problems) |  |  |  |  |  |  |  |  |  |  |
| *Slight problems* |  |  |  |  | 3.535 (3.829) | 0.244 |  |  |  |  |
| *Moderate problems* |  |  |  |  | **11.884* (12.457)** | **0.018** |  |  |  |  |
| *Severe problems* |  |  |  |  | **12.843* (13.673)** | **0.016** |  |  |  |  |
| *Unable* |  |  |  |  | 8.313 (9.728) | 0.070 |  |  |  |  |
| Pain/discomfort (ref: No pain) |  |  |  |  |  |  |  |  |  |  |
| *Slight pain* |  |  |  |  |  |  | 0.946 (0.493) | 0.916 |  |  |
| *Moderate pain* |  |  |  |  |  |  | 2.402 (1.176) | 0.073 |  |  |
| *Severe pain* |  |  |  |  |  |  | **3.029* (1.630)** | **0.039** |  |  |
| *Extreme pain* |  |  |  |  |  |  | 2.731 (2.273) | 0.227 |  |  |
| Anxiety/depression (ref: Not anxious) |  |  |  |  |  |  |  |  |  |  |
| *Slightly anxious* |  |  |  |  |  |  |  |  | 2.124 (0.915) | 0.080 |
| *Moderately anxious* |  |  |  |  |  |  |  |  | 1.633 (0.748) | 0.285 |
| *Severely anxious* |  |  |  |  |  |  |  |  | 1.945 (1.048) | 0.217 |
| *Extremely anxious* |  |  |  |  |  |  |  |  | **17.434 (13.666)** | **<0.001** |
| ^a^ See model (4) in Table 6 for selection of explanatory variables. Participants are grouped by region: Birmingham; Cardiff; Hertfordshire; Leeds; Leicester; London; Newcastle; Oxford; Salford. Complete cases are used. Odds ratios, standard errors, and *P*-values are reported to three decimal places.  ^b^ Statistical significance: * *P*<0.05; ** *P*<0.01; ^¶^ *P*<0.001.  **Abbreviation:** Co.: coefficient; LC: Long COVID; ref: reference; SE: standard error. | | | | | | | | | | |

# Sample of quotes from free-text response

| **Box 1** Select sample of quotes organised by themes |
| --- |
| **Theme 1: Pressure to maintain work performance**  “I'm on an extended phased return […] but under pressure to get back to 30 hours [….] Work has taken over my life [….] I don't do anything else really so that I can manage work. I struggle with concentrating and find that the more I do the worse my symptoms are.”  “Cannot work as hard as I did before due to fatigue and forgetfulness. If I wasn't self employed, I would have sure been fired as I doubt I could do a 9-5 job.”    **Theme 2: Concern over career prospects**  “Able to do limited teaching and admin, not able to do research within reduced work hours. Unable to do research on which retention and promotion depend.”  “Reduced responsibility and hours. Time off has led to reduced experience and will delay promotion prospects. Reduced ability to work will impact future prospects.”  **Theme 3: Financial pressure even in employment**  “Working phased return at home, struggling to get back to full time working; threat of being forced to accept part time work will have a huge financial impact; currently being paid full time rate but fear this cannot last.”  “I work full time in the NHS 37.5 hours a week, I am in the process of reducing my hours to 24hrs a week, due to my Long Covid. I don’t know how financially I will cope.” |
